# Supplementary material for: Poor adherence to TB diagnosis guidelines among under-five children with severe acute malnutrition in central India: A missed window of opportunity?
Source: PLoS One. 2021 Mar 12;16(3):e0248192. doi: 10.1371/journal.pone.0248192 (PMC7954324; doi:10.1371/journal.pone.0248192)
Supplement: S1 Annex — (DOCX) [file pone.0248192.s001.docx]

**Annexure 5:**

**Key informant Interview/Interview guide**

**Name of the participant:**

**Designation:**

**Date of Interview:**

**Interview start / end time:**

**Name of the Interviewer:**

After a brief introduction to the participant regarding the purpose of the interview, the PI/Investigator will take informed written the consent for the interview. Written informed consent will also be requested for audio recording

1. There is diagnostic algorithm for Facility Based Management of Children with Severe Acute Malnutrition Operational Guidelines at NRC & its collaboration with RNTCP programme has been initiated for quite some time…………… What do you think are the positive points about this diagnostic algorithm?

*PI will use the flowchart to describe the diagnosis and treatment pathway for presumptive /confirmed TB patients along with attrition at various levels.*

1. NRC should be screening for TB to all the admitted children as per diagnostic algorithm, Why are all SAM children not being screened? [Probe: programmatic, patient-level] i.e. trainining issues, poor understanding/ambiguity of diagnostic algorithm, feasibility etc…
2. In screening process, why there is overemphasis on Mountoux & CXR? Why there were hardly any serious efforts to obtain the biological specimen of suspected child? [Probe: programmatic, patient-level]
3. Is there any delay in testing and reporting of results at the NRC? If yes, why is it so? [Probe: programmatic, patient-level]
4. Do you feel there is not enough coordination between NRC & DMC level, if yes why? Can you please suggest few measures to improve it.
5. Are there any reasons regarding non / late initiation of treatment for TB cases? [Probe: programmatic, patient-level]
6. What are the operational issues involved in this: both at provider level and patient level?
7. How can we further improve the identification of TB cases at NRCs?
8. Additional remarks, if any?

*PI/Investigator will complete the interview by acknowledging the time spared by the participant from his/her busy schedule. He will also give a summary of the notes taken and confirm the same from the participant.*
