# Supplementary material for: Poor adherence to TB diagnosis guidelines among under-five children with severe acute malnutrition in central India: A missed window of opportunity?
Source: PLoS One. 2021 Mar 12;16(3):e0248192. doi: 10.1371/journal.pone.0248192 (PMC7954324; doi:10.1371/journal.pone.0248192)
Supplement: S3 Annex — (DOCX) [file pone.0248192.s003.docx]

**FGD#1 NRC Staff Sagar**

Date 16/05/2018

Start: 1.45 PM    End 2.42 PM

Number of Participants 7

Interviewer: Akash Ranjan Singh & Ambar Kumar

Study participants demographic detail

| Sr No | Name | Age/Sex | Designation | Work experience |
| --- | --- | --- | --- | --- |
| 1 | XXXXXX | 34/F | FD Bina | 9 Years |
| 2 | XXXXXX | 39/F | FD Sagar | 10 Years |
| 3 | XXXXXX | 42/F | FD Malthon | 5 Years |
| 4 | XXXXXX | 34/F | FD Gadakota | 8 Years |
| 5 | XXXXXX | 30/F | FD Shahgarh | 5 Years |
| 6 | XXXXXX | 28/F | FD Khurai | 4 Years |
| 7 | XXXXXX | 26/F | FD Deori | 4 Months |
| FD* Feeding demonstrator | | | | |

ARS: After explaining them the purpose of the study, we obtained their consent both for taking part in the discussion as well as for the audio recording of the discussion. As you are aware of the purpose of the admission of a child at NRC. We screen the child for certain diseases and try to feed the baby appropriately so that baby gains the weight during its stay. In order to do that, we empower the mother on right feeding practices.

As all of you are working in the NRC's for quite a long time thus, we want to understand from you, how you screen the children for tuberculosis once they admitted to the NRC? As it is one of the diseases that should be screened in every child admitted to the NRCs.

FDs: As the baby admitted in the NRC, we use to question the parents whether any member of their family having cough of long duration, second Moutoux test will be performed, then the screen under CbNAAT. If the child turns out positive for Moutoux and/or CbNAAT it will be put on treatment.

The child is screened with the X-Ray also. After going through the report of above mentioned  3 tests, the doctor will decide whether to start DOT. One thing I have observed, these children (With TB disease) won't gain weight until they were put on DOT. As they started DOT, they immediately start gaining weight, no matter how rich diet & counseling we used to provide, they won't gain weight unless DOT started.

ARS: Can U please explain it?

FDs: Yes Sir!  you can appreciate the weight gain right from day one of treatment (DOT). The child becomes active, open eyes if was lethargic.

We had one child (admitted at NRC), discharged yesterday only, had diarrhoea for 6 days or so, once he was put on DOT, it immediately subsided. It immediately controls infections.

ARS: Would u like to add something on this Mam? (pointing to the FD’s from rural areas)

FD: We used to screen with Moutoux, by examining redness around, used to start DOT.

AK: So, by observing Moutoux result, you start DOT?

FDs: Sometimes used to go for CXR also if advised by the doctors.

We used to ask the history of long duration cough in family members of the children like mother, father or grandparents or neighbours. As the disease might have transmitted to the child through them.

ARS: I want to clarify this as the history related to TB is of two types, one is suggestive to TB in child itself and second is the history of contact to Tb.

FDs: For example; one child was living with Bua, as mother died (must be from TB only), so the history of contact to the family members of Bua need to be asked.

Mamo: It seems that FDs were not aware the History suggestive to TB, however, they were very much aware of how to ask the history of contact.

AK: Whether you consider cough of long duration, TB?

FDs: We use to ask some other history also, like associated fever etc. we ask them to test their sputum to rule out TB. If it turns out TB, they were advised to complete six-month course of treatment.

AK: This complete history you use to take from every child. This is really good practice. Suppose any member of the family had TB 5 yrs back, completed the t/t for 6 months, will you consider the child having a positive history of contact?

FDs: Not Sure!!!

ARS: One thing I would like to know from you all, what are the investigations child should undergo, once he is admitted to the NRC?

Let me clarify myself, we go for a set of tests (to rule out TB) in different children admitted to the NRC, are you aware of any guideline for the screening of TB among children admitted at NRCs?

FDs: (Many at the same time) Mountoux…. And maybe CXR and CbNAAT.

ARS: Whether every child undergoes for CbNAAT? If so then what is the sample we will send for CbNAAT?

FDs: We use to take a sample from the stomach, by putting a tube through the nose.

ARS: Do you take Gastric Aspirate from every child?

FDs: Yes we try to…

ARS: Do all of you take Gastric Aspirate from every child?

FDs: No……Nobody is there in the hospital who can do it? We have never been trained for this.

ARS: Except Sagar NRC, Please tell me what are the tests expected for every child admitted to the NRCs, no matter whether it is being done or not?

FDs: (Silence…………………)

ARS: Ok. Who elicits the h/o related to TB from parents/ mother?

FDs: Family h/o of Tb, h/o f contact, productive cough, …..

AK: to whom you consider “contact”e.g. dada-dadi, nana-nani who lives separately but suffered from TB any time in past?

FDs: To whom child is living with…not sure whether to take dada-dadi, nana-nani in consideration

ARS: Have anyone of you received any training related to screening/diagnosis of TB at NRCs?

FDs: No, we have not received any training pertaining to screening/diagnosis of TB at NRCs?

ARS: Are you aware of the fact that there are two types of h/o. One h/o contact to the TB patients to know the potential source of infection and another ho suggestive of TB in the patients?

FDs: Maybe weight loss, cough. Not sure about others ….

ARS: Whether you were taught about this any time in past?

FDs: No, not at all!

ARS: Let's come to the investigations part. Please tell me, whether Mountoux rest is performed at NRC’s or is done only on some places?

FDs: Yes, We also go for CXR!

ARS: Would u manage to perform CXR for every child?

FDs: (Chorus) Some said yes and most of them said no… its all depends upon the doctor, whether to send CXR or not. For the child below 6 months, we go for CXR. What happened most of the time is, those children turned out positive with the Mantoux are only tested with the CXR. Except for Sagar NRC, it is the routine practice in all other NRCs (of Sagar district).

ARS: Ok, apart from h/o, moutoux, CXR is there anything else need to be done?

FD: In our place, we perform neither (Moutoux & CXR).

ARS: ok the scenario is, as child admitted to NRC, you take h/o, ANM performs Mountoux then result of mountoux interpreted by the ANM. On the basis of mountoux result, doctor used to decided whether to go for CXR or not.

Ok at which day of admission child used to be examined by the doctor for the first time?

FDs: First day itself. That too for overall screening, not particularly for TB.

ARS: Once you are done with the ho, mountoux & CXR do you go for any other test?

FDs: No sir! (6 out of 7) we wont go for any other test if it (mountoux & CXR) come out positive, we facilitate the child to DMC, ask them to register the child and start the treatment.

So, any other test you use to perform before starting the t/t? like Sputum for AFB, CbNAAT, Gastric aspirate, LN biopsy etc.

FDs: No! (5 out of 7). We use to perform these tests to mothers of the admitted children irrespective of their cough status, not to the admitted baby. As you know it's not very easy to obtain samples from the child. At some places, we use to perform CXR of the mother also.

AK: How the moutoux test is performed, I understand this is not primarily your job but to understand your perspective?

FDs: The intradermal injection is administered to the forearm with 5 TU. We make a circle around the site. The result is seen between 48to 78 hr after injection. The test is interpreted positive if redness of more than 5 mm appears from the site.

ARS: Whether u measure redness or induration (Ubhara hua part)?

FDs: No the induration needs to be measured not erythema (lalwala part.

ARS: Positive mountoux as you say more than 5 mm, what does it mean?

FDs: It means a baby has the infections of the TB, we use to take the baby to doctor and start the t/t/ (ATT)

Mamo: Once the mountoux is positive for the child, is perceived by the workers (maybe also by the doctors because of operational feasibility of other tests) that it is sufficient indication to start the t/t.

ARS: Is there any incidence you remember when the sample of sputum for AFB, gastric aspirate or CbNAAT was sent to confirm the TB?

FDs: No! (6 out of 7) we have not seen any such incidence.

ARS: OK, once the baby is diagnosed as positive for TB, then do you perceive any problem in starting of t/t

FDs: No, it started immediately after registration with the DMC, the box of dot is given to the AWW of their area, they will supervise the treatment. After the discharge from NRC, the mother is provided strip needed for 1 week.

ARS: From where you use to start t/t at NRC?

FD: We use to get loose packets from DMC.

ARS: Do you ask for compliance with TB treatment during their fup visits?

FD: No, we don’t, it is the responsibility of programme staff. However, we shall be held responsible if the child doesn’t gain weight.

ARS: Do anyone of you feels any problem in the initiation of treatment even after the diagnosis written by the doctor as our finding in Quantitative part suggest it take a long time to register the patient (long time interval between the date of admission at NRC to date of registration in RNTCp)

AK: In addition to the tests performed by you does the TB workers perform any tests to confirm the diagnosis?

FD: No they never perform any test by themselves. Whatever we perform at NRC, what our doctors write, they start their treatment (They won't take any initiative from their own to confirm the TB)

ARS: Are you aware of Isoniazid prophylactic therapy (IPT) for children having sputum pos contacts in their homes?

FDs: No

ARS: How many of you have heard of it?

FDs: (Only 2 nods…..)

ARS: Do you perceive any problem in coordination between NRC & RNTCP in an order to register & start of t/t?

FDs: Once the doctor wrote to start the treatment, we send the patient to the DMC, they immediately register & start the treatment. But, once the child discharge from NRC we don’t know their compliance to DOT. Even during the fup visits to the NRC we usually not ask about the compliance to DOT.

ARS: Yes! Very right mam, even we have found the same thing in our first phase of the study (quantitative phase). Almost half of the child who was identified at the NRC as TB patients, we don’t have any record (of completion or they are still on treatment). So, in programme whatever is not in record means has not been done.

ARS: Ok fine! Let me show you a graph, it suggests among the all admitted children at NRC only 2% children have undergone all five screening test, approximately 7% children were diagnosed as TB. Out of those who diagnosed, we have treatment records (completion/continue) of only 41% children? What do you think about it?

AK: (Mamo) Barring Sagar NRC, the facility for sputum & gastric aspirate is not available anywhere (in Sagar district). There is over-reliance on Mountoux & once the child turns out mountoux positive then only he is tested for CXR.

FDs: We don’t have any facility equipment for gastric aspirate

AK: Don’t you have any funds available to manage those as these are not very costly. Maybe if you have done it, it is not mentioned in your record. Have you written in case record ‘what samples have been sent’?

FDs: No we don’t have any. We don’t write this as don’t have any column to mention CbNAAT

ARS: There is column mentioned in case of record as “any specific test”. You can/suppose to mention it there only.

Mamo: After the introduction of new records, it was not taught what to be mentioned where)

ARS: Any feedback/suggestion to improve the scenario?

FDs: Our ANMs & We need proper training, what needs to be done in an order to screen TB in children admitted at NRCs. Facilities like NG tube should be available. The child needs to be tracked till the t/t completion (6 months).

ARS: How can u track the child as you have different ID & TB registration no is different. Can we have integrated number for both systems? Can we write TB registration no to our record, once the child put on treatment?

FDs: Yes, that will be fine.

ARS & AK: Thanks for your time & cooperation!!

**KII#1 Staff nurse NRC Sagar**

Date: 16/05/2018

Start: 2.50 PM End 3.12 PM

Name: XXXXXX 37 year/F Work experience: 11 months

Interviewer: Akash Ranjan Singh & Ambar Kumar

ARS:How you screen the SAM children for TB, admitted at NRC?

ANM: We use to ask them whether they have TB disease in the family, any one who is living with or around (neighbour) havig TB or cough of long duration.

ARS:Mam, these are the questions pertaining to the History of contact. Do you ask any history sugeetive of TB in child itself?

ANM: Yes sir we do ask whether child is having cough of more than 3 weeks , having fever for long duration in addition to history of contact like any one from house or neighbour had TB or prolong cough. After obtaining this history, we test the child with mountoux & CbNAAT.

ARS: Please tell us the sequence of events, once child admitted to the NRC?

ANM: Once the child admitted to the NRC, after registration, we ask history of contact to Tb, history suggestive of TB in the first day it self. Then we go for Mountoux test on day one or day 2 of admission. Then, child undergo for physical examination by the Pediatrician preferably on day one. Moutoux is interpreted by myself only after48-72, on the basis of that we start the treatment of Tb for child. Occasionally, madam (Pediatrician) start t/t on the basis of Physical examination also. However, now for quiet some times, we used to send at least one sample of CbNAAT for every child after obtaining the sample from Gastric Aspirate of early morning.

AK: Fine, Mountoux is done by your self only so Mam can u please tell us how you test the child for mountoux?

ANM: We use 0.1 ml TU (5 Tu strength) we inject sub cutaneous in fore arm of the child & make a circle around the injection. We read the result after 2 days, by measuring the redness (not induration) by scale, if it is more than 5 mm we decleare it positive.

*Lal wala bhag scale se napte hain, ubhra hua nahin napte, yadi wah 5 mm se jyada hai to usko positive mante hain.*

ARS: How you document the result of Mountoux test?

ANM: We use to write it in file as well as in case record for every child

*Mamo: However in our quantitative part we found the mountoux result was mentioned on case record for only a proportion of children.*

ARS: What you do after getting mountoux result/

ANM: We show it to the Mam (paediatrician), she use to start DOT, the daily dose regimen.

AK: How you take Gastric aspirate?

ANM: The on-duty nurse of night ensure that child shouldn’t get any thing after 4 am. Then we take sample in morning around 7.00am by Ng tube. Obtaining sample is difficult in older children [say more than 2 years]. It is relatively easier for younger children. We try to take 2 samples but as of now we ensure that we obtain at least one sample of gastric aspirate. As you see some times we don’t have NG tube, sometimes it is very difficult to counsel the parents & obtaining their consent for it. Nevertheless, we still manage them most of the time.

ARS: Do you perceive any problem in registration & initiation of DOT for diagnosed cases of TB?

ANM: No, not at all, once the diagnosis is made by Madam, we use to send the child to DMC for registration. They (DMC staff), use to register them immediately and start the t/t after 1 week through loose pouches till the child remain admitted in the NRC. Once child is discharge, the (treatment) box is provide to the children may be through ASHA or AWW of his village.

ARS: Would you manage to fup the child their after?

ANM; No, we couldn’t fup child once he is discharged from the NRC. Even during the 4 fup visits, we hardly could manage him to ask about the compliance about the treatment, forget about the end of treatment. Even the DMC staff also not used to inform us about the treatment status of the child after the end/completion of treatment.

ARS: Have you go for CXR of the child?

ANM: After the result of Mountoux, madam used to decide whether the child should undergo CXR or not? If she wrote we use to do it. However for quiet some times every child udergo CXR & CbNAAT. If parent give us in writing that they don’t want to put Ng tube inside the child’s nose, then only we cant do it. We use to counsel them, it is free & very important for the child. Sometimes they understand and comply sometimes they don’t. Parents used to afraid of nasal bleeding and all, sometimes they curse us also. Even when we ask history of Contact in any family member, they becomes very angry.

AK: Very right mam, it is the problem we also use to face.

ARS: How do you manage the EP TB suspects?

ANM: Among EP TB cases mostly we get Node ones (Tubercular lymphadenitis). We investigate them as per the order of paediatrician mam, mostly by mountoux, CXR & CbNAAT. We can not go for FNAC and all.

ARS: What about the abdominal TB? If child complain pain in abdomen for long have you manage to investigate him (through USG & all)?

ANM: No sir! Since I am here in last 11 months we have neither find any such child nor investigated through USG. However I have listen that in previous years, mam had send quiet a few patients for USg fro NRc.

ARS: Any suggestion or feedback to improve the screening of Tb at NRC?

ANM: No sir

ARS & AK: Thanks for your time & patience.

**KII#2 DTO Sagar**

Date 17/05/2018

Start: 1.46 PM    End 2.16 PM

Name: XXXXXX 49Year/M Designation: DTO Sagar Work experience: 10 Yr

Interviewer: Akash Ranjan Singh & Ambar Kumar

ARS: After explaining them the purpose of the study, we obtained their consent both for taking part in the discussion as well as for the audio recording of the discussion. As you are aware of the purpose of the admission of a child at NRC.

AK: the child screen at NRC, turn out positive for TB, would be sent to the DMC (district RNTCP) to start tt. How do you start tt for different age group of children?

DTO: We have the chart for different doses according to weight band, pasted all over. It helps us to decide the exact dose of the drugs according to new daily dose regimen. Previously it was Intermittent regimen PC13 & 14. To remove confusions, we decide the dose according to the chart for a given weight of the child.

AK: In our first phase of the study we found that for most of the NRCs, once the child turns out positive for Mountoux test, was put on ATT. Though, at Sagar NRC they send for CbNAAT universally but rest all NRC start ATT on & the only basis of Mountoux test. What do you say about this?

DTO: Sir, There is Guideline for the screening of child at NRC. They should go with that only. A Child should undergo Mountoux test, CXR, Sputum & CbNAAT. If child is positive only for Mountoux test, rest is negative, they should reconsider EP TB.

AK: Yes! We try to find out, in the year 2017, no EP TB case was recorded from NRCs?

DTO: As it is mentioned in programme two type of Tb, one is microbiologically confirmed TB another empirical TB. Once paediatrician decides empirical TB we have to start ATT.

AK: Do you recommend them to put efforts to get microbiologically confirm TB rather making diagnosis as empirical TB & starting ATT straight away.

DTO: Yes in the programme it is mentioned that all possible efforts should be made to make microbiological confirm TB.

AK: But, in children, it is the swelling over neck (Tubercular lymphadenitis) is the most common type of TB.

DTO: If we receive any EP sample we use to process them through CbNAAT.

AK: Do you receive any such samples?

DTO: I can not say specifically about pediatric samples but we receive EP samples of adults on regular basis, even from pvt practitioners.

AK: Do you insist the paediatrician not to start ATT on the basis of Mountoux test, please go for microbiologically confirm TB?

DTO: It is already in place at NRC, for every admitted child we go for CXR, sputum, Cbnaat.

AK: Sir do you receive samples of children? How many samples you receive for each suspected Child

DTO: We receive 2 samples. I am not sure whether we could manage to receive 2 samples from every child.

AK: I find here, only one sample is sent from here also (NRC Sagar), I understand there is practical problems in that…

DTO: No, I need two samples only when first sample is not processed like spillage etc. As now we are practising Universal DST, so for every TB patients needs to be screened for DR/RR TB. For second-line LPA we will send the samples to Bhopal as if it comes out INH resistant the treatment regimen changes. It has started in recent past, as it was not there in 2017.

ARS: Ok fine! Let me show you a graph, here you can see, very few children were there who have gone through all 5 screening as per the guideline. If we see the proportion of children, admitted at NRCs of Sagar & Sheopur, only 1.7% had underwent all 5 screening tests?

AK; It is very likely that few more children would have screened but not been documented. This is the problem everywhere. Isn't it?

ARS: The workers posted at NRCs are at the critical position to deal the children & their parents. If they send the child to DMC, the programme (RNTCP) staff, don’t verify anything they register the patient and start the t/t straightway. Are they trained? As we were talking to your FDs, there was the consensus that either they have not received the training or has no quality check.

DTO: No Sir, They have been trained regularly. You can check the dates also.

Mamo: Once paediatrician decides programme don’t explore any further; a missed opportunity

ARS: Sir, once the child diagnosed at NRC, There is no further exploration/verification by the programme staff?

DTO: It all depends upon pediatrician, whatever he decided, we follow accordingly. We never argue with them, as they are specialist & trained enough to decide. Yes sometimes it may happen, that they may not be aware of the programme guidelines but anyway, these are the guidelines only. So whatever they decide with their clinical acumen we have to rely upon that. Nevertheless, I believe the programme guidelines are crystal clear and everybody should stick with them.

ARS: Sir this problem is more so with the NRC staff, not of RNTCP?

DTO: This problem is for both in fact for all, we have to sort it out with the collaboration betweenall. We have all equipment under RNTCP.

ARS: Any suggestion/feedback to improve screening of Tb at NRC?

DTO: Yes indeed we are having certain problems like inability to perform CbNAAT for all children, ideally, every child should be screened, parents of children should also have been screened. Screening & tt of SAM children, Children of PLHIV parents are more challenging. We have all the facility, the major challenge is the motivation of the staff.

ARS: Thanks for all your time & support.

*Mamo: Denial of problems, however, had insight of the real challenges,*

*More emphasis on training (process indicator), when we ask about outcome he drag us again on process****.***

**FGD#2 TBLT & TBHV**

Date 17/05/2018

Start: 01.05 PM    End 1.40 PM

Number of Participants 7

Interviewer: Akash Ranjan Singh & Ambar Kumar

Study participants demographic detail

| Sr No | Name | Age/Sex | Designation | Work experience |
| --- | --- | --- | --- | --- |
| 1 | XXXXXX | 36/F | TB LT | 2 Years |
| 2 | XXXXXX | 32/M | TB LT | 2 Years |
| 3 | XXXXXX | 33/M | TB HV | 9 Years |
| 4 | XXXXXX | 28/F | TB LT | 4Years |
| 5 | XXXXXX | 40/F | TB HV | 10 Years |
| 6 | XXXXXX | 35/M | TB LT | 13 Years |
| 7 | XXXXXX | 58/M | TB LT | 28 Years |
| TB LT: TB laboratory technician, TB HV: TB health visitor | | | | |

After explaining them the purpose of the study, we obtained their consent both for taking part in the discussion as well as for the audio recording of the discussion. As you are aware of the purpose of the admission of a child at NRC.

We are here to see what are the problem areas & what can be the feasible solutions of those, in context of diagnosis of TB at NRCs in local setting & available resourses?

ARS: Can U please tell us what is being done to screen TB at NRCs?

TBLT: At most of the places the screening of TB is based on Mounux test only. We take History related to TB (suggestive) & history of contact, but, in many cases we may not mention it in the case records. Then on the basis of Mountoux test, doctors use to decide what other tests need to be done. If he (doctor) suggest, child will undergo CXR & the sample for CbNAAT is also sent especially in Sagar. As in Sagar we have 2 CbNAAT machines, one at hospital and another at meical college.

However the situation (the protocol for screening) is different at different NRCs. At few places, even the Mountoux test is not been performed on regular basis because the centers are oftenly stock out. At few NRC hospitals, the x ray machine does not work every now and then.

ARS: Ok, Can U pleas tell us what is the diagnostic guideline for the diagnosis of TB at NRc, as following points will be there to ask in History, Physical Examination & there might be few tests? I understand the situation will be very different from one to another NRc, so I request you all, describe the situation of your own NRC & DMc

TBLT; Yes sir! indeed the situation is very different from one to another NRC, but history suggestive to TB, history of contact are usually asked for every child. At most places the mainstay of diagnosis is Mountoux test, but at few NRCs we are not able to do it on regular basis as oftenly it is stock out. The decision of CXR and other test like CbNAAT is taken by the doctors, nevertheless, every child undergo for CbNAAT admitted at Sagar NRCs but for other places as it mainly depends on the wisdom of the doctor and feasibility of the tests. At places we don’t even have paediatricians posted at NRCs. In such cases the decision making is difficult for *Sukh Rog ya Sukhi ka rog what we call in Bundelkhandi* for the SAM child. At most places, the availability of paediatrician is patchy and MO find it difficult to diagnose/decide, so the children are send to Sagar (NRC) for further evaluation.

ARS: As you said sir every child admitted at NRC should undergo history taking (both for symptoms suggestive of TB & contact), Physical examination, Mouttoux and CXR. Can u manage to do it for all?

LT: No sir we cannot do all these tests for every child. History (of Tb & contact) is asked and mentioned on the case record (NRC) barring this the most feasible test is mountoux so the decision of treatment (for ATT) is taken on the basis of this only. At places, the CXR is also norm but not at all NRCs. At Sagar NRC, even we manage to perform CbNAAT by Gastric Aspirate of almost every child since last few months.

At our place, once the mountoux is done the decision (of treatment) is solely based on that as no other tests are available.

ARS:What does mountoux is suggestive of? As the t/t is started on the basis of this so there is disese?

TBLT: Most of TB HV/LT knows it suggestive of Tb infection but there is no way (operationally feasible), but to start TB.

AK: Once the patient sent to you after tests been done at NRC to start the treatment, could you manage to verify the information and is there any serious efforts are made by the staff present at the DMC to make microbiological confirm TB?

TBLT: You see sir the situation is different at different places. But by and large, the decision to start of treatment is based on Mountoux test only at most places. As we don’t have facility for CXR, CbNAAT and more importantly the leadership of paediatrician/MO, who can facilitate the all diagnosis, guide us what is to be done. Considering all this the mainstay of diagnosis of TB is mountoux barring Sagar NRC.

Some times we used to suggest MO/paediatrician for CXR, CbNAAT but at last what ever he says it goes.

ARS: Could you manage to start the ATT immediately after the diagnosis by MO/Pediatrician?

TBLT: Yes sir we start the treatment immediately, at Sagar we give the ATT through loose pouches, at rest places we give the boxes to patient or to ASHA of that particular village whom the patient belongs to. Then ASHA worker will monitor the treatment regimen.

But, there is a NRC , Malthon, where we couldn’t find any positive (for TB) child since last couple of years. At Deori we don’t have regular supply of Mountoux, it is stock out every now and then.

ARS: From our quantitative data analysis, we found that less than 50% cases (Children diagnosed as TB at NRC) had recorded outcome.

TBLT: ASHAs are poor in documentation as they are educated up to or less than 8^th^ standard. They struggle to maintain proper documentation [mostly in english]. We use to maintain their documentation. Some times they give Continuous phase at initial stage only and vice vesa.

ARS: Who is suppose to supervise them?

STS: We are suppose to visit the patients with in 2 months of registration but its just not possible for us as few of us has almost 800 villages under us and we don’t have any TB HV (except for Bina), so it is not possible for us to supervise them all.

ARS & AK: Any feedback/suggestion

TBLT & STS: IPT sould be available in liquid form & their has to be transport or courier mechanism in place to transport the samples from DMC to DTC.

ARS: Thanks!

**FGD#3 STS Sagar**

Date 17/05/2018

Start: 12.10 PM    End 12.40 PM

Number of Participants 7

Interviewer: Akash Ranjan Singh & Ambar Kumar

Study participants demographic detail

| Sr No | Name | Age/Sex | Designation | Work experience |
| --- | --- | --- | --- | --- |
|  | XXXXXX | 35/M | TB HV | 9 Years |
|  | XXXXXX | 48/M | STS | 16 Years |
|  | XXXXXX | 43/M | STs | 2 Years |
|  | XXXXXX | 43/M | STs | 16 Years |
|  | XXXXXX | 45/M | STS | 16 Years |
|  | XXXXXX | 43/M | STS | 13 Years |

After explaining them the purpose of the study, we obtained their consent both for taking part in the discussion as well as for the audio recording of the discussion. As you are aware of the purpose of the admission of a child at NRC.

ARS: Can you please tell us, how the screening of TB is being done at NRC among admitted children?

LTs: At most of the places in Sagar district, the diagnosis of Tb at NRc is mainly based on Mountoux test. Yes, the NRC staff take the history suggestive to TB, history of contact also. Few of the children also undergo CXR, CbNAAT, but by and large, Mountoux is the mainstay of the diagnosis.

At our hospital & NRC, there is no mountox test was available for quiet some times. So we have not performed any mountoux test since last couple of years. Their is the same scenario in of neighbouring NRCs also.

ARS & AK: Please tell us who & how the diagnosis of TB is made in admitted children of NRCs

LTs; Sir, we take history suggestive of TB, history of contsct for contact tracing, ANM performs mountoux [test], then what tests need to be doone, is decided by the treating physician or Medical officer. As, at few NRCs we don’t have the services of paediatrician. Rarely they (pediatricians) ask for CXR, Sputum for AFB (in case baby can produce sputum). Then he decides whether to start DOT or not. However in Sagar (NRC) we are doing CbNAAT for almost all the children admitted at NRC since last quiet a months. As you asking for entire year 2017, I am not sure about time but as of now we are doing it universally despite of the fact that the NRC staff/ANM is struggling to get NG tube to perform Gastric aspirate of every child.

Now we are also screening all of SAM children for TB through sputum for AFB.

ARS & AK: Once the child is send to you after diagnosis what do you do? Do you verify the history, his of contact etc.

LTS: After the diagnosis by paediatrician, as soon as child come to us, we use to register them & start the DOT. As long as child is staying at NRC, treatment is provided by the FD of NRc then after the discharge, the pachet of drugs is taken by the ASHA worker of their village. Then she use to continue the treatment.

Some times we ourself verify the history/diagnosis written over the case record of the patient but by and large whatever is written by the doctor, we do nothing except to go accordingly.

ARS & AK: In our previous part of study we found that, among the children (admitted to NRC) who were diagnosed for TB, the outcome is known for less than half of the children? Can u all please tell us, what might be the reason for that?

LTS; Once the packet (DOT regimen) is handed over to the ASHA workers, they are suppose to supervise the treatment. They use to do that. But, there might be the chance that they may not have been documented it in the card. As you know, the card is in English, and most of the ASHa workers are educated up to or less than 8^th^ standard. They [ASHAs] are also overburdened with the work related to maternal & child health. They get assured honorarium from there. Our programme is not in their priority as either they do not get or get very late they get honorarium related to TB work.

ARS: Is house visit of patient is also expected by the TB HV & STS?

TBLTs: Yes indeed it is expected that every patient should be visited by the STS with in 2 months after the registration, but as you see our area is very big, we are understaffed as most of the STS are in charge of more than one TU. As of now we have only one TB HV that too in urban area so its not possible for them to visit and supervise the treatment of every patients.

ARS: What about the transport mechsnism for samples? Is there any courier mechanism is place?

STS: At few places its functional but most of the rural areas are devoid of transport mechanism/courier services to transport the sample from periphery to DMC/DT. Most of the time we use to ask either the patient/attendant to take the sample to DMC & submit it.

ARS & AK: Any suggestion or feedback

TBLT: Sir, you see we are here to work , please give us training, support (adequate man power) we can do everything.

ARS: AK: Thanks for your support & time.

**KII#3 Paediatrician Bina**

Dr XXXXXX 59 Yr/M Work experience: 9 years

Date 17/05/2018

Start time: 4.20 pm End time: 4.53 pm

Interviewer: Akash Ranjan Singh & Ambar Kumar

After explaining him the purpose of the study, we obtained their consent both for taking part in the discussion as well as for the audio recording of the discussion.

ARS & AK: As you are aware of the purpose of the admission of a child at NRC. How you use to screen the children admitted to the NRCs?

Pd: Any child admitted to the NRC, I need to find the cause of Malnutrition (SAM), as TB is one amongst them. Myself use to take history suggestive of TB, history of contact i.e. from where it has come to the patient need to be find out. Then I order Moutoux, CXR and Physical examination to see whether the child has enlarged cervical Lymph nodes. We use to write for Gastric aspirate but our sister (ANM) don’t do this. Here the contact history is important i.e; whether any person in house or in neighbourhood having/had cough of long duration. For me its very difficult to ask someone having sputum positive TB in contact of more than 24 hours to the child.

We [people of rural area] have very less awareness about the TB. People see swelling over child’s neck for long but ignore it for long. The elderly had cough for long duration, no body take care of it, in fact the cot of elderly are taken out from house in night so that the sleep of house members don’t get disturb. Usually children are in contact of their grandparents as they have more affinity with them. And, both are immuno-compromised states so more likely to get infeted from one another. In my experience working at NRc, the child turn out positive for TB in 90-95% cases when there is certain/established history of contact.

In our Bina, its very difficult that someone will accept that he is having TB (disease). People use to say it *Moti mahraj.* The course of disease is describe as “for that many days I am suffrringfrom Moti Mahraj, it will go out in that many days”. These are the cases very likely that turn out positive for TB disease either by Sputum/Gastric Aspirate.

ARS & AK: Who intetpret the results of screening tests

MO: ANM does it, myself interpret it, the challenge here is to motivate ANM for Gastric Aspirate, she has so additional duties in NICU, Swasthya Shivir etc. and even she is not motivated enough to do this. Myself do remaining all things,

I believe most of the non responders in NRC are those children who are misdiagnosed (mostly for TB) If I found any non responder use to send then to SMTU Aiims Bhopal.

AK: Do u manage to follow the child at the end of ATT?

MO: No, rather I would like to have those records from the TB programme. It will be nice if they update me about the t/t oucome of child diagnosed & started ATT from NRC.

AK: In our quantitave phase found that most of the children diagnosed from your NRC are microbiological confirm, otherwise rest all the NRC have more number of empirical TB cases.

MO: Yes sir, we only struggle last year when we don’t had X Ray technician, otherwise we try to screen the child through diagnostic algorithem (all five essential components), not merely Mountoux etc. But, I am overburdened with other things also.

ARS & AK: Any suggestion/feedback

MO: Non-responders in NRC, need to be workup again at the end of NRC stay.

All the children diagnosed for TB should get daily dose regimen, even yesterday a child was provided alternate dose regimen, which I opposes.

ARS & AK: Thanks for your time & support.
