# Supplementary material for: Poor adherence to TB diagnosis guidelines among under-five children with severe acute malnutrition in central India: A missed window of opportunity?
Source: PLoS One. 2021 Mar 12;16(3):e0248192. doi: 10.1371/journal.pone.0248192 (PMC7954324; doi:10.1371/journal.pone.0248192)
Supplement: Attachment — Submitted filename: PONE-D-20-30406_reviewer comments.docx [file pone.0248192.s004.docx]

**PONE-D-20-30406_reviewer comments**

- Overall: This is an important study to show missed opportunities of screening, diagnosing, and treating TB in children admitted for SAM in India. Please see below for specific recommendations for improvement. Additionally, my main suggestion would be to expand the scope of the paper (title, etc) to include not only TB screening, but also TB diagnosis and treatment in your cohort. This is covered in your results nicely, and would make the paper much stronger and comprehensive if it focuses on TB diagnosis and TB treatment initiation (in addition to TB screening).
- Cover page:
  - Please correct the title. It appears that “To, The Editor the full title…” was accidentally copy and pasted. Please only list the full title.
  - Key words: also include “TB screening” as a key word
- Title:
  - Recommend including the main findings/message in the title as well. How was it a missed opportunity? Let your read know the main findings/message of the TB screening in the Title.
- Abstract:
  - Please fix various typographical and grammar errors in the abstract (e.g. extra words, spaces, unnecessary quotation marks, etc)
  - In Methods, include the time period of the study (dates of review)
  - Results: in addition to # screened, please also include # diagnosed with TB, # treated for TB, and # finishing treatment/cured (and # deaths/LTFU) of those 3230 admitted. Also please include results of the gastric aspirates mentioned in the Background (how many had gastric aspirates, how many had positive results….as well as any other TB diagnostic test among those 3230).
- Introduction:
  - Lines 78-79, please make it clearer that the % of reported TB in children was only 6%. As written it is not very clear if that is the intended message. Also, you can delete the “There are enough reasons…” sentence, as your next sentence is sufficient (if you slightly modify it).
  - Lines 102-104. Other than gastric aspirate, are there any other recommended TB screening for children with SAM? For example, TB screening questions, score chart, CXR, TST/IGRA, etc? Please mention the entire recommended TB screening protocol for children with TB….or if it is only gastric aspirate, please mention that specifically.
- Methods
  - Lines 117-120: when listing numbers, either use a comma for thousands (e.g. 8,465) or remove the space between the thousands and hundreds place (8465). Having a space (“8 465”) makes it more difficult to read.
  - General settings section: you mention both SMTUs and NRCs…but then your paper only focuses on NRCs? Need more clarification between the two, and why only NRCs examined in the study (what about the SMTUs??)
  - Line 149: Please clarify the exact dates of 2017 reviewed (e.g. 1^st^ January to 31^st^ December)
  - Lines 151-153: more details needed on how health care workers were selected for interviews. You need to show your reader that you took steps and precautions to avoid and minimize selection bias and other biases. More details needed to clearly how you ensured “maximum heterogeneity in the responses.”
  - Data analysis section: the main concern is that your paper’s title focuses on “TB Screening” (which you explained the five steps of doing that in the methods), but then your main output is only microbiologic confirmation. These are two different things….screening vs confirmation of disease. Thus, your main output is not actually a screening output (if screening was done or not in the children). Rather, your main output is microbiological confirmation of TB disease in these children. Very different things. And as you mention in your Introduction, confirmation of TB in young children (especially when SAM) is very difficult and rare.
- Results
  - Qualitative phase section: please include results of all of the screening techniques you mention (i.e. of those screened for XYZ, how many screened positive). Of the Mantoux test, physical exam, suggestive symptoms, CXR, history of contact….we need to know how many screened positive for each of those.
    - Also, please then list how many of the positive screens (and how many of the children with SAM) were diagnosed with TB, and how many started treatment.
  - Line 225: unclear what “infective training” refers to. Need more specifics. Lack of confidence on TB symptoms, reading CXR, score charts, Mantoux testing, sputa extraction? Need more details on the gap.
  - Line 230: is there also reluctance to sputum induction? Or is one method preferred over the other (which is very important information to share!!). which method is accepted more?
- Discussion
  - Lines 282-286. For these five recommendations, can you offer any background or evidence base (citations) that these are effective strategies for your setting? They all seem reasonable, but would strengthen the paper if you can show such interventions have been effective at other settings in India, or globally.
  - Lines 294-297: you can remove these from discussion. These were already covered in Methods and Results. Focus instead on main messages of the study (main take away points)
  - Line 300-301: you mention “availability of diagnostic equipment” here…but in results, this was not mentioned and not listed as a challenge from the interviews. Therefore, can not mention it here unless it is referenced above in results.
- Figures/Tables
  - Figures 2 and 3 are excellent, and show the entire TB treatment cascade. I would focus more on highlighting these tables in the text, and focusing on the “leakage” and issues at each step!
  - Again, your title/paper focuses mostly on TB screening…but you have excellent data along the screening, diagnosis, treatment steps. I would highly recommend you expand the scope of title and paper to include these as well (and not limit it to just screening)
  - Table 1
    - Clarify that “More than 1” actually is only “1-5 years old”
    - Residence: remove this as 84-99% didn’t have it recorded (thus not useful)
      - Same for SES of family (if not recorded, not useful to include)
    - In Table 1, can you also include TB screening (the 5 items) to show how many of the children had each of those 5 screens, and then how many had positive screens of those 5
  - Table 2: please also include the % in ( ) after each number. The first three columns are missing the (%) and only list N
  - Both tables: put your abbreviations footnote below the table (separate) and not as a final row within the table.
