# Supplementary material for: Poor adherence to TB diagnosis guidelines among under-five children with severe acute malnutrition in central India: A missed window of opportunity?
Source: PLoS One. 2021 Mar 12;16(3):e0248192. doi: 10.1371/journal.pone.0248192 (PMC7954324; doi:10.1371/journal.pone.0248192)
Supplement: Attachment — Submitted filename: Response to reviewer comments_V4.docx [file pone.0248192.s007.docx]

*************************************

**RESPONSE TO COMMENTS**

Review Comments to the Author

Reviewer #1:

Comment 1: Overall: The authors have done an excellent job addressing prior review comments and concerns, and the updated manuscript is much improved with a more comprehensive and cohesive message. There are still multiple minor concerns (listed below) that need addressed, but overall the manuscript is vastly improved.

Response: Thank you for the constructive comments. We have carefully responded and addressed your second round of comments.

Comment 2: Cover Page - Abstract:
o Please fix various typographical and grammar errors in the abstract (e.g. extra spaces around “( SAM)”), etc, unnecessary quotation marks, etc)

Response: Thank you for appreciation. Two authors have gone through the manuscript again and corrected the grammar and type errors.

Comment 3: In Results, of the 87 will documented outcomes, please also list of those 87 how many had good outcomes (completed treatment, cured, etc) and how many had poor outcomes (died, LTFU, relapse, etc).

Response: Thank you for suggestion. As the qualitative phase did not explore the reasons for these poor outcomes, we have decided to restrict the results to how many completed treatment and how many did not have a record of their treatment outcome. We hope this is fine. We have done this so that the focus of the abstract remains on TB screening and barriers around it. The line in the revised manuscript now reads as follows

“The treatment outcome was favourable (cure or treatment completed) for 70(31%) and not recorded for 121(54%).” (Page 2 line 49-50 of revised manuscript with track changes)

Comment 4: Abstract
o As above, please include the favorable/unfavorable outcomes of the 87 with recorded outcomes

Response: Thank you for suggestion.

As the qualitative phase did not explore the reasons for these poor outcomes, we have decided to restrict the results to how many completed treatment and how many did not have a record of their treatment outcome. We hope this is fine. We have done this so that the focus of the abstract remains on TB screening and barriers around it. The line in the revised manuscript now reads as follows

“The treatment outcome was favourable (cure or treatment completed) for 70(31%) and not recorded for 121(54%).” (Page 2 line 49-50 of revised manuscript with track changes)

Comment 5: Introduction:
o Line 71, when mentioning “As per guidelines,…” please specify which guidelines (India national, WHO, India NTP, etc)

Response: Thank you for suggestion. We made the changes (Page 3 line 148 of revised manuscript with track changes)

Comment 6: Methods
o Page 4, line 103: please remove the extra space around “ ; “ typo

Response: Thank you for suggestion. We made the changes (Page 4 line 186 of revised manuscript with track changes)

Comment 7: Page 5, line 130: Please describe why only Sagar district had qualitative phase (why not also qualitative phase in Sheopur as well?).

Response: Thank you for your query. The reason was Concerned staff members were not available in Sheopur district because of NHM workers strike during the qualitative study period (June 2018) and limited funds were available under the project, hence only one district was selected. (Page 5 line 214-216 of revised manuscript with track changes)

Comment 8: Page 6, line 137-138: it is mentioned a mobile-based questionnaire was used in Feb-May 2018….however, this paragraph is describing the quantitative phase. Was a questionnaire and mobile app used during the quantitative phase? Or for the qualitative phase?? Also, the quantitative phase extracted data from 2017…but the mobile-based questionnaire took place in 2018…need to clarify this date discrepancy.

Response: A mobile-based questionnaire was used to retrieve the quantitate phase of data. The data pertaining to Jan-Dec 2017 was retrospectively retrieved from Feb – May 2018. After analysis of quantitative data, qualitative phase of study was performed in June 2018.

We have clarified in study design that the quantitative phase was a descriptive study involving secondary data collection. Under study population we have clarified that we included under-five children with SAM that were admitted in 2017. Under data collection, we have clarified that secondary data was extracted from the records during Feb-May 2018. Qualitative data collection was done in June 2018. We hope this is fine.

Please see Page 6 lines 235-237, 241 in revised manuscript with track changes.

Comment 9: Likewise, for the qualitative (interviews) phase…please list the dates these were conducted in the “Data collection and sources of data” section.

Response: Thank you. The qualitative phase of data collection was done in June 2018. (Page 6 line 241 of revised manuscript with track changes)

Comment 10: Results
o Page 8, line 188: please again list the dates of the study (to help remind your readers) at the start of the first sentence, e.g. “Between 1st Jan and 31st December 2017, a total of 3230 SAM…”

Response: Thank you for suggestion. We would not like to repeat the dates again in the results sections as this will be a repletion and may cause reader fatigue. These details have been clarified under study population in methods section. We hope this is fine.

Comment 11: Page 8, paragraph of lines 192-196. Need to use alternate terms other than “positive” when discussing symptoms suggestive of TB and chest xray results. It is not commonly terminology to say “positive” for these two screening techniques. Instead, you can say “202 were found to have symptoms suggestive of TB” and “159 had chest xray results suggestive of pTB” respectively.

Response: Thank you for suggestion. We made the change (Page 8 line 374-379 of revised manuscript with track changes)

Comment 12: Page 8, line 195: please recheck the calculations for the CXR %. 159 is not 1% of 1220 for CXR. Likewise, it is unclear what 485 is 2% of (what is the denominator here?)

Response: Thank you for suggestion. We have made the changes and clarified the same. We made the change (Page 8 line 377-378 of revised manuscript with track changes)

Comment 13: Page 8, lines 197-200: interestingly, as written, your yield of CBNAAT (24%) is much lower than your yield of smear (44%). This is the opposite of the commonly accepted data that Xpert has HIGHER yield than smear in children with TB. If correct, you need to discuss why smear performed so much better than CBNAAT in this cohort (which is highly unusual).

Response: Thank you for suggestion. In the context of poor record keeping and poor coverage of TB screening, these two percentages are not comparable. We tried to explain this in the first para of discussion section (Page 12 line 757-759 of revised manuscript with track changes)

Comment 14: Also, please re-word what is meant by “28 children were labeled as clinically confirmed TB.” By WHO definition, “confirmed” means that a sample was found to have MTb (e.g. sputum). Clinically-diagnosis of TB (without “confirmed”) means that a diagnosis of TB was made based on S/Sx, physical exam, CXR, TST, etc…but without confirmation by Xpert/Culture/smear. If you remove “confirmed” here, it will be ok.

Response: Thank you for suggestion. We made the change. (Page 8 line 383-384 of revised manuscript with track changes)

Comment 15: Page 8 line 201, please add a descriptor to the “Of 3230,” (e.g. “Of the 3230 diagnosed with TB,…”)

Response: Thank you for suggestion. We made the change. (Page 8 line 385 of revised manuscript with track changes)

Comment 16: Page 8, line 205-206. Is any information available on the 6% of child with SAM and TB who were not started on ATT? Died? LTFU? Why not started? Also, for the 87 with outcome data, please also include if it was favorable or unfavorable outcome.

Response: Thank you for suggestion. We couldn’t explore the reason for the same. As there was no record available at DMC near to NRC. It seems poor coordination between NRC & RNTCP staff. Ideally, these children should have been notified during their stay at NRC itself and same should have been mentioned in their Case sheet of NRC. We included the outcome for those it was documented. (Page 8 line 390-391 of revised manuscript with track changes)

Comment 17: Page 8, line 206-208: the total in this line equals 209, but not the 223 of children with TB.

Response: Thank you for suggestion. We made the change. (Page 8 line 389-391 of revised manuscript with track changes)

Comment 18: Page 10: Figure 2 is not included (only the title and the footnotes are currently in the clean revisions manuscript). Please include the actual Figure 2.

Response: As per PLOS ONE guidelines, after we cite a figure in result narrative, we are required to insert the figure title and footnote. However the Figure itself has to be submitted separately in the form of an image file (tiff format). In other words, the figure is not part of the manuscript file. However, the reviewer can access the figure file in the submission pdf.

Comment 19: Discussion
o Page 12, line 284, please remove the comma after “Even though,” (not needed)

Response: Thank you for suggestion. We made the change. (Page 12 line 756 of revised manuscript with track changes)

Comment 20: Page 12, line 286, please add “According to our findings,” before the “Adherence to initial assessment…” sentence (as this is what your findings showed)

Response: Thank you for suggestion. We made the change. (Page 12 line 760 of revised manuscript with track changes)

Comment 21: Page 13, line 330-331, would suggest removing the “particularly affective and psychomotor domain” portion of sentence, as neither of those was studied or reported in your current study. Better to keep your conclusions focused and linked to your study findings (and not introduce too many new ideas suddenly in the discussion).

Response: Thank you for suggestion. We made the change. (Page 14 line 841 of revised manuscript with track changes)

Comment 22: Line 332: need a period at the end of the sentence (after references 40-44).

Response: Thank you for suggestion. We made the change. (Page 14 line 843 of revised manuscript with track changes)

Comment 23: Line 333-334: would rephrase that “when samples were collected, a high proportion were collected via gastric aspirate” since overall, a low percentage of children with SAM had any sample collected; but of the few with samples it was done via gastric aspiration (i.e. preferred method).

Response: Thank you for suggestion. We have actually revised the sentence as follows “Low coverage of collection and testing of gastric aspirate might be due to the lack of assured specimen transport mechanism to the CBNAAT testing facilities at the district headquarters” (Page 14 line 843-844 of revised manuscript with track changes)

Comment 24: Line 335-337: would remove this sentence about transport of specimens, as again, this was not a focus of this study, nor mentioned in Results section, nor was MDR part of this study.

Response: Thank you for suggestion. We made the change in this sentence as per above comments, didn’t removed the sentence, we hope reviewer will find it OK.. (Page 14 line 843-844 of revised manuscript with track changes)

Comment 25: Line 347, change to “this present study documented poor adherence to initial TB screening, assessment, and TB diagnostic guidelines…”

Response: Thank you for suggestion. We made the change. (Page 14 line 854 of revised manuscript with track changes)

Comment 26: General suggestion: throughout the discussion, the theme of putting blame on the NRC in-charge (i.e. a top-down approach) is prevalent throughout. While it may have been reported during the qualitative interviews, I would caution you and the authors on your persistent focus of putting such blame on the in-charge….since both TB and SAM care for children requires a team approach and teamwork mentality to provide the highest quality of comprehensive care. Each clinician and NRC team member should be empowered with responsibility and motivated to have ownership of their actions and of the care they provide. I worry a bit that the recurrent “blame the in-charge” message could be inappropriately used as a way to absolve other team members of blame and of suboptimal care. I would highly suggest you consider alternative way to re-phrase your findings so that instead of singular blame, the message is to better inspire team work and foster personal responsibility to provide the best care possible (even in challenging work situations with limited resources)…rather than simply blaming and pointing the finger at the in-charge.

Response: Thank you for suggestion. We change the phrase to “poor team skills” in fig 2.

Comment 27: Figures/Tables
o Figure 1 and Figure 2: the image quality is poor/blurry. Please use a higher definition image (higher quality) that is legible. Also, there is a blue rectangle pixilation in the upper right (typo? Screen shot artifact?) that needs removed.

Response: Thank you for suggestion. We made the change. (Fig 1 & 2)

Comment 28: There is also a superscript “1” in the “Identified by community & others” box that has no definition at the bottom of the figure to show what the “1” refers to

Response: Thank you for suggestion. We remove this. (Fig 1 & 2)

Comment 29: For the % used, please be consistent with your decimal places. Some % have no decimal point (no tenths of a percentage), while others do use a decimal point (and a tenth of percentage). Also, several boxes do not have any (%) listed (e.g. “referred/died”. Please have consistency throughout the figure.

Response: Thank you for suggestion. We made the change. (Fig 1 & 2)

Reviewer #2:

Comments to the Author:

Point 1: In the method section (Page 5, Line 111), the author did not explain how to use the 5 criteria to diagnose tuberculosis. Please check it.

Response: Thank you for suggestion. If microbiological diagnosis is not feasible, the in-charge pediatrician or Medical Officer can make a clinical diagnosis depending on the initial assessment using 5 criteria and further investigations of examination findings. (Page 5 line 195-208 of revised manuscript with track changes)

Point 2: In the result section, the description of table1 and figure1 was illogical, the author should rearrange the structure of description.

Response: Thank you for suggestion. We made the changes. (Table 1 & Fig 1)

Point 3: In the result section (Page 8, Line 192), the description “of them 178 (7%) tested positive” was inconsistent with description of Mantoux test in table 1. The author should correct it.

Response: Thank you for pointing our mistake. We made the correction. (Page 8 line 374 of revised manuscript with track changes)

Point 4: The total column of “Time-gap between NRC admission and DOT initiation” in the Table 1 was not necessary. Please check it.

Response: Dear Reviewer, we have deleted it from table 1

Point 5: In the result section (Page 12, Line 278), the full name of “FNAC” should be given when it first appeared in the text. Please check it carefully.

Response: Thank you for suggestion. Actually, we have removed FNAC as our quote does not discuss about it. (Page 12 line 750 of revised manuscript with track changes)

Point 6: In the footnotes of figures, we could not find “AFP” and “SSM” in the figure 1 and figure 2. The author should check it carefully.

Response: Thank you for suggestion. We made the changes
