# Supplementary material for: Poor adherence to TB diagnosis guidelines among under-five children with severe acute malnutrition in central India: A missed window of opportunity?
Source: PLoS One. 2021 Mar 12;16(3):e0248192. doi: 10.1371/journal.pone.0248192 (PMC7954324; doi:10.1371/journal.pone.0248192)
Supplement: Attachment — Submitted filename: Response to reviewer comments.doc [file pone.0248192.s005.doc]

*************************************

**RESPONSE TO COMMENTS**

**EDITORIAL STAFF COMMENTS**

COMMENTS
Please ensure that your manuscript meets PLOS ONE's style requirements, including those for file naming. The PLOS ONE style templates can be found at

<https://journals.plos.org/plosone/s/file?id=wjVg/PLOSOne_formatting_sample_main_body.pdf>

<https://journals.plos.org/plosone/s/file?id=ba62/PLOSOne_formatting_sample_title_authors_affiliations.pdf>

RESPONSE

Thanks for your comment. We tried to best of our capacity to meet the journal’s guideline. The revised manuscript is as per journal requirements

COMMENTS
2. Please include captions for your Supporting Information files at the end of your manuscript, and update any in-text citations to match accordingly. Please see our Supporting Information guidelines for more information: <http://journals.plos.org/plosone/s/supporting-information>.

RESPONSE

Thanks for your comment. We tried to meet the journal’s guideline related to Supporting documents. The revised manuscript is as per journal requirements

COMMENTS
3. When reporting the results of qualitative research, we suggest consulting the COREQ guidelines: <http://intqhc.oxfordjournals.org/content/19/6/349>. In this case, please consider including more information on the following: 1) the number of interviewers, their training and characteristics, 2) please provide the interview guide used 3) additional information regarding the number of participants in each focus group and 4) Information regarding the pilot of the guides and whether any modifications were made.

RESPONSE

Thanks for your comment. We have incorporated the desired information in the manuscript. Please refer to lines 183-2045 of revised manuscript with track changes.

COMMENTS
4. In ethics statement in the manuscript and in the online submission form, please provide additional information about the patient records/samples used the qualitative aspect of the study. Specifically, please ensure that you have discussed whether all data/samples were fully anonymized before you accessed them.

RESPONSE

Thanks for your comment. The data pertaining to the study participants (quantitative as well as qualitative) were fully anonymized. We have mentioned this in ethics statement and also in online submission form.

COMMENTS
5. Please include your tables as part of your main manuscript and remove the individual files. Please note that supplementary tables (should remain/ be uploaded) as separate "supporting information" files

RESPONSE

Thanks for your suggestion. We have made this correction in the manuscript as per suggestion.

COMMENTS
6. We note you have included a table to which you do not refer in the text of your manuscript. Please ensure that you refer to Table 1 and 2 in your text; if accepted, production will need this reference to link the reader to the Table.

RESPONSE

Thanks for pointing our mistake. We have made this correction in the manuscript as per suggestion. Table 1 and 2 have been cited in the text.

COMMENTS
7.We note that [Figure(s) 1] in your submission contain map images which may be copyrighted. All PLOS content is published under the Creative Commons Attribution License (CC BY 4.0), which means that the manuscript, images, and Supporting Information files will be freely available online, and any third party is permitted to access, download, copy, distribute, and use these materials in any way, even commercially, with proper attribution. For these reasons, we cannot publish previously copyrighted maps or satellite images created using proprietary data, such as Google software (Google Maps, Street View, and Earth). For more information, see our copyright guidelines: <http://journals.plos.org/plosone/s/licenses-and-copyright>.

We require you to either (1) present written permission from the copyright holder to publish these figures specifically under the CC BY 4.0 license, or (2) remove the figures from your submission:

1.    You may seek permission from the original copyright holder of Figure(s) [1] to publish the content specifically under the CC BY 4.0 license.

We recommend that you contact the original copyright holder with the Content Permission Form (<http://journals.plos.org/plosone/s/file?id=7c09/content-permission-form.pdf>) and the following text:

“I request permission for the open-access journal PLOS ONE to publish XXX under the Creative Commons Attribution License (CCAL) CC BY 4.0 (<http://creativecommons.org/licenses/by/4.0/>). Please be aware that this license allows unrestricted use and distribution, even commercially, by third parties. Please reply and provide explicit written permission to publish XXX under a CC BY license and complete the attached form.”

Please upload the completed Content Permission Form or other proof of granted permissions as an "Other" file with your submission.

In the figure caption of the copyrighted figure, please include the following text: “Reprinted from [ref] under a CC BY license, with permission from [name of publisher], original copyright [original copyright year].”

2.    If you are unable to obtain permission from the original copyright holder to publish these figures under the CC BY 4.0 license or if the copyright holder’s requirements are incompatible with the CC BY 4.0 license, please either i) remove the figure or ii) supply a replacement figure that complies with the CC BY 4.0 license. Please check copyright information on all replacement figures and update the figure caption with source information. If applicable, please specify in the figure caption text when a figure is similar but not identical to the original image and is therefore for illustrative purposes only.

The following resources for replacing copyrighted map figures may be helpful:

USGS National Map Viewer (public domain): <http://viewer.nationalmap.gov/viewer/>

The Gateway to Astronaut Photography of Earth (public domain): <http://eol.jsc.nasa.gov/sseop/clickmap/>

Maps at the CIA (public domain): <https://www.cia.gov/library/publications/the-world-factbook/index.html> and <https://www.cia.gov/library/publications/cia-maps-publications/index.html>

NASA Earth Observatory (public domain): <http://earthobservatory.nasa.gov/>

Landsat: <http://landsat.visibleearth.nasa.gov/>

USGS EROS (Earth Resources Observatory and Science (EROS) Center) (public domain): [http://eros.usgs.gov/#](http://eros.usgs.gov/)

Natural Earth (public domain): <http://www.naturalearthdata.com/>

RESPONSE

Thanks for suggestion. We have removed the figure 1 & renumbered the remaining ones.

COMMENTS
8. Please ensure that you refer to Figure 2 in your text as, if accepted, production will need this reference to link the reader to the figure.

RESPONSE

Thanks for pointing our mistake. We have made this correction in the manuscript as per suggestion.

COMMENTS
9. PLOS requires an ORCID iD for the corresponding author in Editorial Manager on papers submitted after December 6th, 2016. Please ensure that you have an ORCID iD and that it is validated in Editorial Manager. To do this, go to ‘Update my Information’ (in the upper left-hand corner of the main menu), and click on the Fetch/Validate link next to the ORCID field. This will take you to the ORCID site and allow you to create a new iD or authenticate a pre-existing iD in Editorial Manager. Please see the following video for instructions on linking an ORCID iD to your Editorial Manager account: <https://www.youtube.com/watch?v=_xcclfuvtxQ>

RESPONSE

Thanks for your suggestion. We are providing the ORCID ID of the corresponding author.

COMMENTS
10. Thank you for stating the following in your Competing Interests section:

[None].

Please complete your Competing Interests on the online submission form to state any Competing Interests. If you have no competing interests, please state "The authors have declared that no competing interests exist.", as detailed online in our guide for authors at http://journals.plos.org/plosone/s/submit-now

This information should be included in your cover letter; we will change the online submission form on your behalf.

RESPONSE
Thanks for pointing our mistake, we will completes our Competing Interests on the online submission as “The authors have declared that no competing interests exist."

*******************************************

**REVIEWER COMMENTS**

**REVIEWER 1**

**(Line numbers in our response are with reference to the revised manuscript with track changes)**

COMMENT

Overall: This is an important study to show missed opportunities of screening, diagnosing, and treating TB in children admitted for SAM in India. Please see below for specific recommendations for improvement. Additionally, my main suggestion would be to expand the scope of the paper (title, etc) to include not only TB screening, but also TB diagnosis and treatment in your cohort. This is covered in your results nicely, and would make the paper much stronger and comprehensive if it focuses on TB diagnosis and TB treatment initiation (in addition to TB screening).

RESPONSE
We appreciate the reviewer for his words of encouragement. We have documented the level of initial assessment ,adherence to TB diagnostic guidelines, yield of microbiological confirmation among those undergoing testing. We also looked at treatment initiation and outcome (for the purpose of completing the care cascade). The qualitative part mostly focused on gaps in TB screening. Therefore we have limited our title to TB screening / diagnosis. To make the title more catchy (in line with you next comment), we have revised it as follows

“Poor adherence to TB diagnosis guidelines among under-five children with severe acute malnutrition in central India: a missed window of opportunity?”

COMMENT

Cover page:

Please correct the title. It appears that “To, The Editor the full title…” was accidentally copy and pasted. Please only list the full title.

RESPONSE
Thanks for pointing out the mistake. We have corrected the title.

COMMENT

Key words: also include “TB screening” as a key word

RESPONSE
Thanks for suggestion. We have made the change

COMMENT

Title:

Recommend including the main findings/message in the title as well. How was it a missed opportunity? Let your read know the main findings/message of the TB screening in the Title.

RESPONSE

Thank you. We have incorporated this suggestion and included the main message / finding in the title.

COMMENT

Abstract:

Please fix various typographical and grammar errors in the abstract (e.g. extra words, spaces, unnecessary quotation marks, etc)

RESPONSE
Thanks for suggestion. Two authors have carefully gone through the manuscript and corrected the typo and grammar errors.

COMMENT

In Methods, include the time period of the study (dates of review)

RESPONSE
Thanks for the suggestion. We have included the period of review (Feb-May 2018). Please refer to lines 78-79 and 185-86 of revised manuscript with track changes.

COMMENT

Results: in addition to # screened, please also include # diagnosed with TB, # treated for TB, and # finishing treatment/cured (and # deaths/LTFU) of those 3230 admitted. Also please include results of the gastric aspirates mentioned in the Background (how many had gastric aspirates, how many had positive results….as well as any other TB diagnostic test among those 3230).

RESPONSE
 Thanks for suggestion. We have made the changes. (lines 82-85 of revised manuscript with track changes). We have reproduced the revised section below

“Of 3230 admitted children with SAM, 547(17%) were screened for TB as per guidelines, 223(7%) were diagnosed and 209 (6%) were treated for TB. Of 209 on treatment, outcome was documented for 87 (4%). Among 318(10%) whose gastric aspirate was obtained, 140 (4.3%) were microbiologically confirmed.”

COMMENT

Introduction:

Lines 78-79, please make it clearer that the % of reported TB in children was only 6%. As written it is not very clear if that is the intended message. Also, you can delete the “There are enough reasons…” sentence, as your next sentence is sufficient (if you slightly modify it).

RESPONSE
Thanks for suggestion. We have made the changes. (lines 93-94 of revised manuscript with track changes)

COMMENT

Lines 102-104. Other than gastric aspirate, are there any other recommended TB screening for children with SAM? For example, TB screening questions, score chart, CXR, TST/IGRA, etc? Please mention the entire recommended TB screening protocol for children with TB….or if it is only gastric aspirate, please mention that specifically.

RESPONSE
Thanks for suggestion. , the gastric lavage (preferably) or induced sputum should be obtained for every admitted child in the NRC for the CBNAAT depending on feasibility. In case, the diagnosis of TB cannot be established by this method, the in-charge pediatrician or Medical Officer can also make a clinical diagnosis depending on the initial assessment and further investigations. The screening, diagnosis and treatment of TB for the children admitted at NRC is described in detail under the following section under settings‘’ Care pathway for identification of TB cases among children admitted at NRCs’ (lines 153-171 of revised manuscript with track changes)

COMMENT

Methods

Lines 117-120: when listing numbers, either use a comma for thousands (e.g. 8,465) or remove the space between the thousands and hundreds place (8465). Having a space (“8 465”) makes it more difficult to read.

RESPONSE
Thanks for suggestion. We have made the change as suggested.

COMMENT

General settings section: you mention both SMTUs and NRCs…but then your paper only focuses on NRCs? Need more clarification between the two, and why only NRCs examined in the study (what about the SMTUs??)

RESPONSE
Thanks for pointing this out. The guidelines for the screening, diagnosis and treatment of TB is same at NRC and SMTUs, so for the simplicity of the readers we described the SMTUs as any other NRC in the paper. (lines 144-45 of revised manuscript with track changes)

COMMENT

Line 149: Please clarify the exact dates of 2017 reviewed (e.g. 1^st^ January to 31^st^ December)

RESPONSE
Thanks for pointing this out. We have now clarified this. (line 175 of revised manuscript with track changes)

COMMENT

Lines 151-153: more details needed on how health care workers were selected for interviews. You need to show your reader that you took steps and precautions to avoid and minimize selection bias and other biases. More details needed to clearly how you ensured “maximum heterogeneity in the responses.”

RESPONSE
Thanks for the suggestion. The investigators conducted 3 FGDs, one with NRC staff another with programme staff of district TB control programme who works either at public health institution (PHI) or at Designated microscopic centers (DMC) and one FGD with STS, whose main work is to register the TB patient and make home visits to those patients to ensure the drug adherence. Investigators invited the participants of one rank in same FGD so that the participants feel free to discuss the topic among them. At the same time to ensure maximum heterogeneity in the response we invited at least one participant from every PHI/DMC.

For the KII also we selected one staff nurse, one District TB Officer and one NRC in charge Pediatrician to ensure the maximum heterogeneity in the responses and also to represent all the important stakeholders involve in the screening, diagnosis and treatment of childhood TB among children admitted at the NRCs. (Page 7 line 190-199 of revised manuscript with track changes)

COMMENT

Data analysis section: the main concern is that your paper’s title focuses on “TB Screening” (which you explained the five steps of doing that in the methods), but then your main output is only microbiologic confirmation. These are two different things….screening vs confirmation of disease. Thus, your main output is not actually a screening output (if screening was done or not in the children). Rather, your main output is microbiological confirmation of TB disease in these children. Very different things. And as you mention in your Introduction, confirmation of TB in young children (especially when SAM) is very difficult and rare.

RESPONSE
Thanks for your comment which has provided us invaluable insight regarding the study. The main concern of our paper is poor adherence to TB diagnostic guidelines. For the sake of completion, we have also mentioned how many were microbiologically confirmed among those tested, among total diagnosed (clinical and microbiologically), how were started on treatment and what was the outcome. Microbiological confirmation among those tested cannot be representative of the true picture of its burden because a large number did not undergo tests for microbiological confirmation. We hope this in fine.

As the TB control Programme of India advocates every child admitted at the NRC has to be screened through CBNAAT by obtaining gastric aspirate. As these are the SAM children and TB might be a cause or consequence of SAM.

COMMENT

Results

Qualitative phase section: please include results of all of the screening techniques you mention (i.e. of those screened for XYZ, how many screened positive). Of the Mantoux test, physical exam, suggestive symptoms, CXR, history of contact….we need to know how many screened positive for each of those.

Also, please then list how many of the positive screens (and how many of the children with SAM) were diagnosed with TB, and how many started treatment.

RESPONSE
Thanks for your suggestion to improve the manuscript. The proportion of SAM children underwent different tests for the screening of TB and proportion of children tested positive with respective tests are described in the result section. (line 253-256 of revised manuscript with track changes)

The number of positive screens & number of children diagnosed as TB is been described in result section. (line 257-60)

COMMENT

Line 225: unclear what “infective training” refers to. Need more specifics. Lack of confidence on TB symptoms, reading CXR, score charts, Mantoux testing, sputa extraction? Need more details on the gap.

RESPONSE
Thanks for your suggestion to improve the manuscript. Sorry for the typo error. We have removed the phrase “ineffective training” and clarified it by replacing it with “Lack of training”. (lines 304 to 307 of revised manuscript with track changes)

COMMENT

Line 230: is there also reluctance to sputum induction? Or is one method preferred over the other (which is very important information to share!!). which method is accepted more?

RESPONSE
Thanks for your suggestion to improve the manuscript. The practice of sputum induction is not very common in the studied NRCs. Ideally, sputum induction & gastic aspirate has almost equal yield but due to some reason the practice of sputum induction was not very common in any of the studied NRC. We have discussed

COMMENT

Discussion

Lines 282-286. For these five recommendations, can you offer any background or evidence base (citations) that these are effective strategies for your setting? They all seem reasonable, but would strengthen the paper if you can show such interventions have been effective at other settings in India, or globally.

RESPONSE
Thanks for your suggestion to improve the manuscript. Please refer to lines 394-405 of revised manuscript with track changes (discussion section) where we have addressed this. We have dded additional references from 47 to 53 to justify this.

COMMENT

Lines 294-297: you can remove these from discussion. These were already covered in Methods and Results. Focus instead on main messages of the study (main take away points)

RESPONSE
Thanks for suggestion. We have removed that line and focused on the main take away points. We have specially ensured that we do not repeat the result values in the discussion section.

COMMENT

Line 300-301: you mention “availability of diagnostic equipment” here…but in results, this was not mentioned and not listed as a challenge from the interviews. Therefore, we can not mention it here unless it is referenced above in results.

RESPONSE
 Thanks for suggestion. Availability of diagnostic equipment was one of our recommendations. This was because the overall the extent of testing of gastric aspirate, Mantoux test and chest radiographs was low. Qualitative data showed that poor leadership of in-charge pediatrician/medical officer was one of the reasons for the absence of facility for chest radiograph and CBNAAT (see lines 315-323 of revised manuscript with track changes). We hope this is fine now.

COMMENT

Figures/Tables

Figures 2 and 3 are excellent, and show the entire TB treatment cascade. I would focus more on highlighting these tables in the text, and focusing on the “leakage” and issues at each step!

RESPONSE
 Thanks for appreciation. We tried to describe the highlights of theses tables in result section with leakage & issues at each step. (line 248 to 264 of revised manuscript with track changes)

COMMENT

Again, your title/paper focuses mostly on TB screening…but you have excellent data along the screening, diagnosis, treatment steps. I would highly recommend you expand the scope of title and paper to include these as well (and not limit it to just screening)

RESPONSE
Thanks for appreciation. We have clarified this point before in response to your earlier comment regarding why we have restricted the title to screening. We have revised it mentioning the key finding (as suggested in another comment of yours)

COMMENT

Table 1

Clarify that “More than 1” actually is only “1-5 years old”

**Response**: Thanks for suggestion. We have changed the same in the table (Table 1)

COMMENT

Residence: remove this as 84-99% didn’t have it recorded (thus not useful)

RESPONSE
Thanks for the suggestion. This indicates, poor record keeping and is important to highlight. Residence details are important for tracking patients for treatment related purpose as well. Hence, with your permission, we would like to retain it.

COMMENT

Same for SES of family (if not recorded, not useful to include)

RESPONSE
 Thanks for the suggestion. However, investigators have consensus that this is an important factor which determines the access of NRC services by the community. Despite of this fact, the NRC staff doesn’t even capture it, indicates, poor record keeping. Hence, with your permission, investigators want to retain this.

COMMENT

In Table 1, can you also include TB screening (the 5 items) to show how many of the children had each of those 5 screens, and then how many had positive screens of those 5

RESPONSE
 Thanks for the suggestion, we have added this to the table 1 (Table 1)

COMMENT

Table 2: please also include the % in ( ) after each number. The first three columns are missing the (%) and only list N

RESPONSE
 Thanks for the suggestion, we have added % to the screened and diagnosed column. As admitted column is the denominator, the percentage is not applicable here. (Table 2)

COMMENT

Both tables: put your abbreviations footnote below the table (separate) and not as a final row within the table.

RESPONSE
Thanks for the suggestion, we have changed the table 1 & 2 accordingly (Table 1&2)

******************************************

**REVIEWER 2
(Line numbers in our response are with reference to the revised manuscript with track changes)**

COMMENT

Point 1: In the section of Introduction (Page 10, Line 2), the “6.%” was incomplete information. The author should check it and ensure the accuracy of the information.

RESPONSE

Thank you for the comment. We have clarified it in the revised manuscript. It now reads as follwows.

“Globally in 2017, an estimated 10% of the total diagnosed tuberculosis (TB) patients were children (<15 years) but in India this was six percent among reported TB [1].”

COMMENT

Point 2: In the section of Data collection and sources of data (Page 13, Line 16), the full name of “DMCs” should be given when it first appeared in the text.

RESPONSE

Thank you for the comment. We have provided the full name of DMC. We have gone through the document again and ensured that all abbreviations are mentioned in full on first use.

COMMENT

Point 3: In the section of Result, the format of the number of cases and percentages in Figure 2 should be unified. Besides, some footnotes were inconsistent with the contents in the Figure2 and Figure3.

RESPONSE

Thank you for the comment. We have reviewed as advised and made the corrections

COMMENT

Point 4: Detailed descriptions of tables 1 and 2 cannot be found in the Result section, please check it carefully.

RESPONSE

Thank you for the comment. We have provided detailed descriptions of table 1 and 2 in the results section. Please see lines 248-260 of revised manuscript with track changes.

COMMENT

Point 5: The formats of the statistical tables were not standard, and the contents of footnotes were inconsistent with the contents of the tables.

RESPONSE

Thank you for the comment. In the revised manuscript, We have carefully reviewed the formatting of tables and ensured that they meet the accepted standards. We have carefully reviewed the footnotes of the tables as well.

COMMENT

Point 6: The corresponding percentages were missing after number in Table 2.

RESPONSE

Thank you for the comment. Thanks for the suggestion, we have added % to the screened and diagnosed column. As admitted column is the denominator, the percentage is not applicable here. (Table 2)

COMMENT

Point 7: There were too much description of results in the discussion section. The author should refer to other studies for further discussion.

RESPONSE

Thank you for the comment. In the revised manuscript, we have made sure that we do not repeat the results figures in the narrative text of discussion. We also expanded the discussion by discussing more about – one-third coverage of chest radiograph, why induced sputum was not being used as an alternative to gastric lavage in these settings, looking at other literature (ref 47 to 53) where our recommendations have been found to be useful. Please refer to 349-405 lines of revised manuscript with track changes.

COMMENT

Point 8: There were many spelling errors in the manuscript. The author should check and correct it.

RESPONSE

Thank you for the comment. Two authors have carefully gone through the manuscript again and corrected all the typo and grammar errors. Sorry that this happened in the first place.
