# Supplementary material for: Poor adherence to TB diagnosis guidelines among under-five children with severe acute malnutrition in central India: A missed window of opportunity?
Source: PLoS One. 2021 Mar 12;16(3):e0248192. doi: 10.1371/journal.pone.0248192 (PMC7954324; doi:10.1371/journal.pone.0248192)
Supplement: Attachment — Submitted filename: PONE-D-20-30406_R1 revisions reviewer comments.docx [file pone.0248192.s006.docx]

**PONE-D-20-30406_R1 revisions - reviewer comments**

- Overall: The authors have done an excellent job addressing prior review comments and concerns, and the updated manuscript is much improved with a more comprehensive and cohesive message. There are still multiple minor concerns (listed below) that need addressed, but overall the manuscript is vastly improved.
- Cover Page - Abstract:
  - Please fix various typographical and grammar errors in the abstract (e.g. extra spaces around “( SAM)”), etc, unnecessary quotation marks, etc)
  - In Results, of the 87 will documented outcomes, please also list of those 87 how many had good outcomes (completed treatment, cured, etc) and how many had poor outcomes (died, LTFU, relapse, etc).
- Abstract
  - As above, please include the favorable/unfavorable outcomes of the 87 with recorded outcomes
- Introduction:
  - Line 71, when mentioning “As per guidelines,…” please specify which guidelines (India national, WHO, India NTP, etc)
- Methods
  - Page 4, line 103: please remove the extra space around “ ; “ typo
  - Page 5, line 130: Please describe why only Sagar district had qualitative phase (why not also qualitative phase in Sheopur as well?).
  - Page 6, line 137-138: it is mentioned a mobile-based questionnaire was used in Feb-May 2018….however, this paragraph is describing the quantitative phase. Was a questionnaire and mobile app used during the quantitative phase? Or for the qualitative phase?? Also, the quantitative phase extracted data from 2017…but the mobile-based questionnaire took place in 2018…need to clarify this date discrepancy.
  - Likewise, for the qualitative (interviews) phase…please list the dates these were conducted in the “Data collection and sources of data” section.
- Results
  - Page 8, line 188: please again list the dates of the study (to help remind your readers) at the start of the first sentence, e.g. “Between 1^st^ Jan and 31^st^ December 2017, a total of 3230 SAM…”
  - Page 8, paragraph of lines 192-196. Need to use alternate terms other than “positive” when discussing symptoms suggestive of TB and chest xray results. It is not commonly terminology to say “positive” for these two screening techniques. Instead, you can say “202 were found to have symptoms suggestive of TB” and “159 had chest xray results suggestive of pTB” respectively.
  - Page 8, line 195: please recheck the calculations for the CXR %. 159 is not 1% of 1220 for CXR. Likewise, it is unclear what 485 is 2% of (what is the denominator here?)
  - Page 8, lines 197-200: interestingly, as written, your yield of CBNAAT (24%) is much lower than your yield of smear (44%). This is the opposite of the commonly accepted data that Xpert has HIGHER yield than smear in children with TB. If correct, you need to discuss why smear performed so much better than CBNAAT in this cohort (which is highly unusual).
    - Also, please re-word what is meant by “28 children were labeled as clinically confirmed TB.” By WHO definition, “confirmed” means that a sample was found to have MTb (e.g. sputum). Clinically-diagnosis of TB (without “confirmed”) means that a diagnosis of TB was made based on S/Sx, physical exam, CXR, TST, etc…but without confirmation by Xpert/Culture/smear. If you remove “confirmed” here, it will be ok.
  - Page 8 line 201, please add a descriptor to the “Of 3230,” (e.g. “Of the 3230 diagnosed with TB,…”)
  - Page 8, line 205-206. Is any information available on the 6% of child with SAM and TB who were not started on ATT? Died? LTFU? Why not started? Also, for the 87 with outcome data, please also include if it was favorable or unfavorable outcome.
  - Page 8, line 206-208: the total in this line equals 209, but not the 223 of children with TB.
  - Page 10: Figure 2 is not included (only the title and the footnotes are currently in the clean revisions manuscript). Please include the actual Figure 2.
- Discussion
  - Page 12, line 284, please remove the comma after “Even though,” (not needed)
  - Page 12, line 286, please add “According to our findings,” before the “Adherence to initial assessment…” sentence (as this is what your findings showed)
  - Page 13, line 330-331, would suggest removing the “particularly affective and psychomotor domain” portion of sentence, as neither of those was studied or reported in your current study. Better to keep your conclusions focused and linked to your study findings (and not introduce too many new ideas suddenly in the discussion).
  - Line 332: need a period at the end of the sentence (after references 40-44).
  - Line 333-334: would rephrase that “when samples were collected, a high proportion were collected via gastric aspirate” since overall, a low percentage of children with SAM had any sample collected; but of the few with samples it was done via gastric aspiration (i.e. preferred method).
  - Line 335-337: would remove this sentence about transport of specimens, as again, this was not a focus of this study, nor mentioned in Results section, nor was MDR part of this study.
  - Line 347, change to “this present study documented poor adherence to initial TB screening, assessment, and TB diagnostic guidelines…”
  - General suggestion: throughout the discussion, the theme of putting blame on the NRC in-charge (i.e. a top-down approach) is prevalent throughout. While it may have been reported during the qualitative interviews, I would caution you and the authors on your persistent focus of putting such blame on the in-charge….since both TB and SAM care for children requires a team approach and teamwork mentality to provide the highest quality of comprehensive care. Each clinician and NRC team member should be empowered with responsibility and motivated to have ownership of their actions and of the care they provide. I worry a bit that the recurrent “blame the in-charge” message could be inappropriately used as a way to absolve other team members of blame and of suboptimal care. I would highly suggest you consider alternative way to re-phrase your findings so that instead of singular blame, the message is to better inspire team work and foster personal responsibility to provide the best care possible (even in challenging work situations with limited resources)…rather than simply blaming and pointing the finger at the in-charge.
- Figures/Tables
  - Figure 1 and Figure 2: the image quality is poor/blurry. Please use a higher definition image (higher quality) that is legible. Also, there is a blue rectangle pixilation in the upper right (typo? Screen shot artifact?) that needs removed.
    - There is also a superscript “1” in the “Identified by community & others” box that has no definition at the bottom of the figure to show what the “1” refers to
    - For the % used, please be consistent with your decimal places. Some % have no decimal point (no tenths of a percentage), while others do use a decimal point (and a tenth of percentage). Also, several boxes do not have any (%) listed (e.g. “referred/died”. Please have consistency throughout the figure.
