# Supplementary material for: Poor adherence to TB diagnosis guidelines among under-five children with severe acute malnutrition in central India: A missed window of opportunity?
Source: PLoS One. 2021 Mar 12;16(3):e0248192. doi: 10.1371/journal.pone.0248192 (PMC7954324; doi:10.1371/journal.pone.0248192)
Supplement: Attachment — Submitted filename: PONE-D-20-30406_R2 revisions reviewer comments.docx [file pone.0248192.s008.docx]

**PONE-D-20-30406_R2 revisions - reviewer comments**

- Overall: The authors have done a good job addressing certain comments. However, there are still issues and gaps that need addressed to make the manuscript stronger and flow more logically and coherently. There are also some data discrepancies between results and discussion that need addressed.
- Abstract
  - In Background, you specifically mention “presumptive pediatric pulmonary TB patients” however the title and rest of the abstract simply uses “TB” (without the ‘pulmonary’ preface). Throughout your study, are you only reviewing/reporting on pulmonary TB? Or does your data include all types of child TB (pTB and EPTB)? Whatever the case, either remove “pulmonary” here, or add “pulmonary” throughout so it is clear and harmonized in the paper (“pTB” vs “TB”).
  - In Objective, remove “(2017)” and instead change to “in 2017.”
  - Methods, remove the “Between February and May 2018”, as it is not critical to report when the analysis was done. Rather, it is much more important to know the dates of retrospective review (Jan-Dec 2017). Writing when the study teamed reviewed the 2017 data is unnecessary (and could add to some date confusion of the reader). Instead, you can start the Methods with “An explanatory mixed methods study was conducted to analyze the 2017 child TB data among under-fives with SAM at NRCs in Sagar and Sheopur districts. The NRC records were reviewed,….”
  - Methods, your definition of “adherence to TB diagnosis guidelines” as listed in the abstract is very narrow (i.e. it only focuses on specimen collection). TB diagnostic guidelines also involve clinical history suggestive of TB, physical exam findings suggestive of TB, lack of response to other non-TB treatments, and other non-sputum/molecular tests (e.g. radiography/CXR, IGRA, etc). When assessing “adherence to TB diagnostic guidelines” (yes/no), did you team evaluate all of these diagnostic pathways? Or just whether a specimen for AFB smear and/or Xpert was obtained? If the former, please clarify this in the abstract.
  - Results, in your abstract, you provide no results for the “Operationally, adherence to TB diagnosis guidelines” (yes/no) that was mentioned in your Methods. Please lists the data on what %/N were “yes” for adhered to guidelines. Your results only mention TB screening and gastric aspirates….which are a part/subset of “TB diagnostic guidelines” but are not comprehensive to TB diagnosis.
  - In Results, “poor team skills” as written is unclear and not a commonly used term. I know this is in response to an earlier comment, but needs rephased/rewritten so that it is easily understandable to your audience what you are trying to convey as a challenge. Telling someone “poor team skills” is a challenge is hard to interpret what exactly you mean.
  - Conclusion: needs rephrased. The single sentence as written is difficult to understand. For example, you write “its utilization remains suboptimal” yet the subject of the sentence is NRCs….thus, is NRC utilization suboptimal? Or do you mean that following TB screening and diagnostic guidelines within the NRCs are suboptimal? This conclusion in the abstract needs rewritten to better summarize the findings of your study.
- Introduction:
  - Line 63: here you mention the high rates of EPTB in child TB; to my earlier comment above…is your study only looking at pTB or all types of TB? If only pTB, it seems odd to mention the higher rates of EPTB in children…but then not to analyze EPTB in your cohort. Line 82 specifically mentioned “pediatric pulmonary TB” again. What about children with EPTB? You need to make it abundantly clear if you are only assessing pTB or all types TB in your cohort of under-fives with SAM.
  - Line 76 and line 80. Add appropriate spaces after the period (before “In”).
  - Lines 83-85. Please add in a sentence or details linking the gaps in India’s NRCs to what you aimed to do with your study. E.g. “In order to try and better address and understand the reason for these large TB treatment gaps in children with SAM, our study aimed to estimate the yield…” Otherwise, the final two sentences do not flow well from the rest of your Introduction.
- Methods
  - Line 139, please delete “between Feb and May 2018” as it is not necessary to report when your study team did their data extraction/analysis. What is important is to share the dates the data was extracted from (2017), which you have already done. The 2018 dates could add to date confusion for the audience.
- Results
  - Line 196, add a space after “(6%)”
  - Line 212-213: there are 209 who started TB treatment, but you only have 70 with favorable and 121 not recorded…which is 191 total. What about the other 18 patients (of the 209)?? Your outcomes (since they include “not recorded” should tally the 209 who started ATT
  - Again, I am having trouble on how you are defining “Screened for TB” in your Table 2. It says only 17% screened for TB in this table. But in your text above, it mentions 83% had Mantoux text, 70% were asked about symptoms of TB….and both of these are ways to screen for TB. From your text, it looks like upwards of 70-83% were screened for TB (in one way or another), but your table says only 17%? This is confusing to the reader and needs clarified and addressed. I think your table means to show who had smear or Xpert…which is different from “Screened as per guidelines”, I would recommend removing “screened as per guidelines” and instead write “Underwent smear microscopy or rapid molecular test”
  - Line 261 and section: again, consider different terminology. “poor team skills” is not commonly used, and vague and hard to understand as written. Lack of motivation, lack of ownership, or work avoidance seem better terms to use what I think you are trying to describe.
  - Line 273: I don’t think “ambiguity” is the correct term for the challenge here. As written, it sounds like the way the Indian TB guidelines are written is not clear and ambiguous. Is that the issue? Guidelines are not clearly written? Or is it again a type of “work avoidance” where team members don’t want to have the responsibility of diagnosis and treating TB in children?
  - Line 279: “Non-availability” or “Unavailable” would be more appropriate terms to use here instead of “Non-feasibility”
- Discussion
  - Line 285-286: as mentioned above in Abstract, this first sentence needs rewritten for clarification and ease of understanding. It makes it sound like NRC utilization is suboptimal as written.
  - Line 287: change “coverage” to “implementation”
    - Also, I still struggle to understand how your smear yield is so much better and so much higher than your Xpert/CBNAAT yield. The added text doesn’t help explain this. Can you elaborate more on why this mis-matched performance of AFB smear vs Xpert in your cohort?
  - Line 288: were diagnostic guidelines different? Or do you mean they way they were implemented (or lack of implementation at sites) is what differed? I think you may want to change “followed” to “implemented”
  - Line 300: here you say less than one-third…but in your results (line 198 ) you say 2277/70% were asked about symptoms suggestive of TB. This wide data discrepancy needs to addressed and aligned.
  - Line 309-310: again, you say one-third had CXR and Mantoux test…but in your results (line 196), there were 2665/83% with Mantoux testing. Again, your Results and your Discussion need to align and harmonize.
  - Line 310-311: recommend changing “availability” to “usage” or “implementation” as the availability or stock out of such test are not related to teamwork or motivation (but usage is)
  - In your Discussion, many paragraphs talk about the quantitative findings (and interpreting them). But there really aren’t any paragraphs in your discussion that talk about and interpret the various qualitative (interview) findings of your study that you present in your results. Before jumping into your “The following are our recommendations” paragraph, it would be good to discuss and interpret the findings of your qualitative phases (the interviews) in your Discussion. Doing this would also make “your recommendations” flow more naturally and be derived from the findings in your Results. Likewise, in line 336 you mention how the qualitative phase was “invaluable in complementing”; yet the qualitative findings receive little to no attention in your discussion
  - Line 336: add a spacing before this new paragraph.
- Figures/Tables
  - Figure 1 and Figure 2: the image quality is still very poor/blurry in the built pdf. It is difficult to read due to blurriness, and needs a higher quality image that is legible.
