# Supplementary material for: Poor adherence to TB diagnosis guidelines among under-five children with severe acute malnutrition in central India: A missed window of opportunity?
Source: PLoS One. 2021 Mar 12;16(3):e0248192. doi: 10.1371/journal.pone.0248192 (PMC7954324; doi:10.1371/journal.pone.0248192)
Supplement: Attachment — Submitted filename: Response to reviewer comments.docx [file pone.0248192.s009.docx]

Dear Editor,

We would like to thank the reviewer for their comments. We have made two key changes in the revised manuscript. We now hope that the manuscript meet the satisfaction of the editor as well as the reviewers.

The two key changes are

Based on the comments of reviewer 1, in the revised manuscript we have clarified that we determined the extent of clinical assessment and assessment for microbiological confirmation among under-five children with SAM admitted at NRCs. The number (proportion) undergoing clinical assessment and microbiological confirmation have been presented separately.

We have made minor edits in the Figure files for better clarity and ensured that the content in the figures matches with the results narrative. We have ensured that the figures are clear (not blurred) and meet PLOS ONE requirements.

With regards,

Corresponding author

***********************

**RESPONSE TO REVIEWERS COMMENTS**

**Reviewer #1:**

Comment

 • Overall: The authors have done a good job addressing certain comments. However, there are still issues and gaps that need addressed to make the manuscript stronger and flow more logically and coherently. There are also some data discrepancies between results and discussion that need addressed.

Response: Thank you for the appreciation. We tried our best to address the issues and gaps suggested by the reviewer in an order to improve the manuscript. Based on your comments we have also removed any data discrepancies between result and discussion section.

Comment

• Abstract
o In Background, you specifically mention “presumptive pediatric pulmonary TB patients” however the title and rest of the abstract simply uses “TB” (without the ‘pulmonary’ preface). Throughout your study, are you only reviewing/reporting on pulmonary TB? Or does your data include all types of child TB (pTB and EPTB)? Whatever the case, either remove “pulmonary” here, or add “pulmonary” throughout so it is clear and harmonized in the paper (“pTB” vs “TB”).

Response: Our study intended to include all forms of TB. We have removed “pulmonary” wherever applicable. We have ensured consistency in the revised manuscript.

All children with SAM that are admitted to NRCs were expected to undergo TB screening. This includes assessment for microbiological confirmation (using upfront rapid molecular test, operationally we included sputum microscopy as well) and clinical assessment (history suggestive to TB, history of contact, physical examination, chest x ray & Mantoux test).

Comment

o In Objective, remove “(2017)” and instead change to “in 2017.”

Response: Thanks for suggestion. We made the change. (Line 37 & 95 in Revised Manuscript with track changes)

Comment

o Methods, remove the “Between February and May 2018”, as it is not critical to report when the analysis was done. Rather, it is much more important to know the dates of retrospective review (Jan-Dec 2017). Writing when the study teamed reviewed the 2017 data is unnecessary (and could add to some date confusion of the reader). Instead, you can start the Methods with “An explanatory mixed methods study was conducted to analyze the 2017 child TB data among under-fives with SAM at NRCs in Sagar and Sheopur districts. The NRC records were reviewed,….”

Response: Thanks for suggestion. The cohort of under five children admitted to NRC belonged to 2017 (based on date of admission). Between Feb 2018 and May 2018 the records of these children were reviewed. I think this is important to clarify. Including the time when data extraction was done was also suggested by reviewer 2, we agreed with the suggestions and included this information during revision. We need to give some time of (retrospective) follow up for those admitted in the end of Dec 2017 before we reviewed their data. Hence, we started the review of records in Feb 2018. This is the reason why some children were still under treatment (line 228-29 of revised manuscript with track changes). We have not mentioned Feb-May 2018 in abstract but mentioned it under data collection in main text. We hope this is fine.

The study design reads as follows (lines 101-02 of revised manuscript with track changes)

“An explanatory type of mixed methods study design was used [27]. The descriptive quantitative phase involving secondary data collection was followed by a descriptive qualitative phase.”

The data collection line reads as follows (line 149-51 of revised manuscript with track changes)

“In the quantitative phase, between February and May 2018, information related to screening, diagnosis and treatment was extracted and captured in a mobile based data collection tool [32].”

Comment

o Methods, your definition of “adherence to TB diagnosis guidelines” as listed in the abstract is very narrow (i.e. it only focuses on specimen collection). TB diagnostic guidelines also involve clinical history suggestive of TB, physical exam findings suggestive of TB, lack of response to other non-TB treatments, and other non-sputum/molecular tests (e.g. radiography/CXR, IGRA, etc). When assessing “adherence to TB diagnostic guidelines” (yes/no), did you team evaluate all of these diagnostic pathways? Or just whether a specimen for AFB smear and/or Xpert was obtained? If the former, please clarify this in the abstract.

Response: Thank you. In this revised manuscript we have clarified that we determined the extent of clinical assessment and assessment for microbiological confirmation among under-five children with SAM admitted at NRC. The number (proportion) undergoing clinical assessment and microbiological confirmation have been presented separately. We have consistently mentioned this in the abstract and main text as well. Please see lines 35-36, 46-50, 95-96, 179-83, line 212-21 in revised manuscript with track changes. I am copy pasting below the following from lines 179-83 of revised manuscript with track changes.

“Key analytic outputs were the number (proportion) of children that were clinically assessed (each criteria presented separately) and the number (proportion) that underwent smear microscopy or rapid molecular tests to establish microbiological confirmation. It was operationally decided to include sputum microscopy for the assessment of microbiological confirmation”

Comment

o Results, in your abstract, you provide no results for the “Operationally, adherence to TB diagnosis guidelines” (yes/no) that was mentioned in your Methods. Please lists the data on what %/N were “yes” for adhered to guidelines. Your results only mention TB screening and gastric aspirates….which are a part/subset of “TB diagnostic guidelines” but are not comprehensive to TB diagnosis.

Response: We have removed this statement “Operationally, adherence to TB diagnosis guidelines (yes/no)” in main text. In continuation to our responses above, we have clarified that TB screening included clinical assessment (each criteria presented separately) and assessment for microbiological confirmation (CBNAAT or sputum microscopy). Results also have been presented separately for clinical assessment (each criteria presented separately) and assessment for microbiological confirmation (CBNAAT or sputum microscopy). Please see lines 35-36, 46-50, 95-96, 179-83, line 212-21 in revised manuscript with track changes. We hope this is now clear.

Comment

o In Results, “poor team skills” as written is unclear and not a commonly used term. I know this is in response to an earlier comment, but needs rephased/rewritten so that it is easily understandable to your audience what you are trying to convey as a challenge. Telling someone “poor team skills” is a challenge is hard to interpret what exactly you mean.

Response: This edit in the phrase of the theme (previously it was poor leadership by NRC in charge) was made during prior revisions based on the suggestion by reviewer 2. Hence, we are retaining this phrase ‘poor team skills’. We hope this is fine.

Comment

o Conclusion: needs rephrased. The single sentence as written is difficult to understand. For example, you write “its utilization remains suboptimal” yet the subject of the sentence is NRCs….thus, is NRC utilization suboptimal? Or do you mean that following TB screening and diagnostic guidelines within the NRCs are suboptimal? This conclusion in the abstract needs rewritten to better summarize the findings of your study.

Response: Thank you for the comment. We have revised as follows (line 60-52, 322-24) of revised manuscript

“Though NRCs provided a unique window of opportunity for the screening and management of TB among under-five children with SAM, the utilization of this opportunity remained suboptimal.”

Comment

• Introduction:
o Line 63: here you mention the high rates of EPTB in child TB; to my earlier comment above…is your study only looking at pTB or all types of TB? If only pTB, it seems odd to mention the higher rates of EPTB in children…but then not to analyze EPTB in your cohort. Line 82 specifically mentioned “pediatric pulmonary TB” again. What about children with EPTB? You need to make it abundantly clear if you are only assessing pTB or all types TB in your cohort of under-fives with SAM.

Response: Our study intended to include all forms of TB and their distributions in terms of how they are being diagnosed i.e. microbiologically confirm TB or clinically confirm TB. We have ensured that we mention this consistently throughout the manuscript. However, we didn’t capture how many of the children with TB had extrapulmonary TB and hence, have not mentioned them in the results.

Comment

o Line 76 and line 80. Add appropriate spaces after the period (before “In”).

Response: Thanks for suggestion. We made the change.

Comment
o Lines 83-85. Please add in a sentence or details linking the gaps in India’s NRCs to what you aimed to do with your study. E.g. “In order to try and better address and understand the reason for these large TB treatment gaps in children with SAM, our study aimed to estimate the yield…” Otherwise, the final two sentences do not flow well from the rest of your

Response

We have revised this (see lines 93-98 of revised ma manuscript with track changes, reproduced below)

“In order to understand the TB diagnosis gaps in children with SAM, our study aimed to determine the yield of and adherence to the TB diagnostic guidelines (clinical assessment and assessment for microbiological confirmation) among under-five children with SAM admitted at NRCs of Madhya Pradesh, India in 2017. We also explored the challenges in screening from the health care providers’ perspective.”

Comment
• Methods
o Line 139, please delete “between Feb and May 2018” as it is not necessary to report when your study team did their data extraction/analysis. What is important is to share the dates the data was extracted from (2017), which you have already done. The 2018 dates could add to date confusion for the audience.

Response: Thank you for the suggestion. The cohort of under five children admitted to NRC belonged to 2017 (based on date of admission). Between Feb 2018 and May 2018 the records of these children were reviewed. I think this is important to clarify. This was also suggested by reviewer 2, we agreed with the suggestions and included this information. We need to give some time of (retrospective) follow up for those admitted in the end of Dec 2017 before we reviewed their data. Hence, we started the review of records in Feb 2018. This is the reason why some children were still under treatment (line 230-31 of revised manuscript with track changes). We have not mentioned Feb-May 2018 in abstract but mentioned it under data collection in main text. We hope this is fine.

The study design reads as follows (lines 101-02 of revised manuscript with track changes)

“An explanatory type of mixed methods study design was used [27]. The descriptive quantitative phase involving secondary data collection was followed by a descriptive qualitative phase.”

The data collection line reads as follows (line 149-51 of revised manuscript with track changes)

“In the quantitative phase, between February and May 2018, information related to screening, diagnosis and treatment was extracted and captured in a mobile based data collection tool [32].”

Comment

• Results
o Line 196, add a space after “(6%)”

Response: Thanks for suggestion. We made the change.

Comment

o Line 212-213: there are 209 who started TB treatment, but you only have 70 with favorable and 121 not recorded…which is 191 total. What about the other 18 patients (of the 209)?? Your outcomes (since they include “not recorded” should tally the 209 who started ATT

Response: Thanks for suggestion. We described the treatment outcome for all 209 children with SAM who initiated on ATT. (Line 228 of Revised Manuscript with tract changes).

“Of 209, the treatment outcome was favourable for 70(34%) (cured or treatment completed). Treatment was still going on for 17(8%), outcome was not recorded for 121(58%) and one child failed the treatment”

209 = 70 + 17 +121 +1

Comment

o Again, I am having trouble on how you are defining “Screened for TB” in your Table 2. It says only 17% screened for TB in this table. But in your text above, it mentions 83% had Mantoux text, 70% were asked about symptoms of TB….and both of these are ways to screen for TB. From your text, it looks like upwards of 70-83% were screened for TB (in one way or another), but your table says only 17%? This is confusing to the reader and needs clarified and addressed. I think your table means to show who had smear or Xpert…which is different from “Screened as per guidelines”, I would recommend removing “screened as per guidelines” and instead write “Underwent smear microscopy or rapid molecular test”

Response: Thank you for the suggestion, in Table 2, we have replaced ‘screened for TB’ with ‘assessed for microbiological confirmation’. This is also in line with our previous comment that we have removed “Operationally, adherence to TB diagnosis guidelines (yes/no)” in the main text and presented clinical assessment and assessment for microbiological assessment separately.

Comment

o Line 261 and section: again, consider different terminology. “poor team skills” is not commonly used, and vague and hard to understand as written. Lack of motivation, lack of ownership, or work avoidance seem better terms to use what I think you are trying to describe.

Response: This edit in the phrase of the theme (previously it was poor leadership by NRC in charge) was made based on the suggestion by reviewer 2 during the previous rounds of reviews. Hence, we are retaining this phrase ‘poor team skills’. We hope this is fine.

Comment

o Line 273: I don’t think “ambiguity” is the correct term for the challenge here. As written, it sounds like the way the Indian TB guidelines are written is not clear and ambiguous. Is that the issue? Guidelines are not clearly written? Or is it again a type of “work avoidance” where team members don’t want to have the responsibility of diagnosis and treating TB in children?

Response: This was the perception of the health care providers. CBNAAT was not available in all facilities. The screening and diagnostic guidelines were not explicit regarding what was to be done when certain tests/facilities were either not available or not feasible due to lack of assured specimen transport; therefore the screening guidelines were not consistently followed across the NRCs.

We discussed this again internally. We believe that “non-feasibility of CBNAAT” (previously included as a separate theme) is covered under the description of theme “ambiguity in diagnostic algorithm”. Hence we have deleted the theme “non-feasibility of CBNAAT” from the narrative text as well as Figure 2.

We have clarified this in lines 304-312 of revised manuscript with track changes.

Comment

o Line 279: “Non-availability” or “Unavailable” would be more appropriate terms to use here instead of “Non-feasibility”

Response: We discussed this again internally. We believe that “non-feasibility of CBNAAT” (previously included as a separate theme) is covered under the description of theme “ambiguity in diagnostic algorithm”. Hence, we have deleted the theme “non-feasibility of CBNAAT” from narrative text as well as Figure 2. CBNAAT was not available in all facilities. The screening and diagnostic guidelines were not explicit regarding what was to be done when certain tests/facilities were either not available or not feasible due to lack of assured specimen transport; therefore the screening guidelines were not consistently followed across the NRCs.

Comment

• Discussion
o Line 285-286: as mentioned above in Abstract, this first sentence needs rewritten for clarification and ease of understanding. It makes it sound like NRC utilization is suboptimal as written.

Response: As shared before, we have modified the statement. Thank you for this suggestion. Lines 320-22 of revised manuscript with track changes.

Comment

o Line 287: change “coverage” to “implementation”

Response: Thanks for suggestion. We made the change. (Line 324 in Revised Manuscript with tract changes)

Comment

♣ Also, I still struggle to understand how your smear yield is so much better and so much higher than your Xpert/CBNAAT yield. The added text doesn’t help explain this. Can you elaborate more on why this mis-matched performance of AFB smear vs Xpert in your cohort?

Response: In the context of poor implementation of TB screening (akin to high non response in a survey), the yield in our small non-representative sample cannot be extrapolated to all children admitted at NRC. We clarified this in discussion section (see lines 22-25 of revised manuscript with track changes, reproduced below).

“In the context of poor implementation of TB screening, the yield of smear microscopy and CBNAAT in the small subgroup of children who underwent testing cannot be extrapolated to all the children admitted at NRC (n=3230). The diagnostic guidelines implemented at different NRCs were different”

Comment

o Line 288: were diagnostic guidelines different? Or do you mean they way they were implemented (or lack of implementation at sites) is what differed? I think you may want to change “followed” to “implemented”

Response: Thanks for suggestion. We made the change. (Line 327 in Revised Manuscript with tract changes)

Comment

o Line 300: here you say less than one-third…but in your results (line 198 ) you say 2277/70% were asked about symptoms suggestive of TB. This wide data discrepancy needs to addressed and aligned.

Response: Thanks for pointing this mistake. We made the change. (Line 340-42 in Revised Manuscript with tract changes)

Comment

o Line 309-310: again, you say one-third had CXR and Mantoux test…but in your results (line 196), there were 2665/83% with Mantoux testing. Again, your Results and your Discussion need to align and harmonize.

Response: Thanks for pointing this mistake. We made the change. (Line 350 in Revised Manuscript with tract changes)

Comment

o Line 310-311: recommend changing “availability” to “usage” or “implementation” as the availability or stock out of such test are not related to teamwork or motivation (but usage is)

Response: Thanks for pointing this mistake. We made the change. (Line 352 in Revised Manuscript with tract changes)

Comment

o In your Discussion, many paragraphs talk about the quantitative findings (and interpreting them). But there really aren’t any paragraphs in your discussion that talk about and interpret the various qualitative (interview) findings of your study that you present in your results. Before jumping into your “The following are our recommendations” paragraph, it would be good to discuss and interpret the findings of your qualitative phases (the interviews) in your Discussion. Doing this would also make “your recommendations” flow more naturally and be derived from the findings in your Results. Likewise, in line 336 you mention how the qualitative phase was “invaluable in complementing”; yet the qualitative findings receive little to no attention in your discussion

Response: Thanks for the suggestion. We considered your comments but the authors feel that the discussion section is fine in the present form. We have discussed the quantitative and qualitative findings together. This was an explanatory mixed methods study. The findings of the quan phase were explored indepth (why?) in the qual phase. While interpreting the quan findings in the discussion section, we also side by side discussed the relevant qual findings. Hence, we did not find the need to have a separate qual paragraph in the discussion section. We hope this is fine.

During this revision, we have added relevant heading in the discussion section, this will hopefully; make it easy for the reader. The headings are: key findings, recommendations, strengths and limitations, conclusion

Comment

o Line 336: add a spacing before this new paragraph.

Response: Thanks. We added the space.

Comment

• Figures/Tables
o Figure 1 and Figure 2: the image quality is still very poor/blurry in the built pdf. It is difficult to read due to blurriness, and needs a higher quality image that is legible.

Response: Thanks for pointing our mistake. The image files (Fig 1 and 2) are submitted separately at the time of submission. We have made sure that the image files are clear and meet PLOS ONE requirement. When these image files are merged into the pdf by the manuscript submission website (while the software generates the submission pdf file), it is not uncommon for the figures to become blurred. However, as this comment has been made for the second time by the reviewer, we have remade the figures using MS powerpoint and submitted the more clear image files as figure files. (previously we used insert canvas option in MS word to make the figure files). We have taken this opportunity to make minor edits in Fig 1 and 2 so that they are consistent with the narrative.

Once accepted, the journal editorial office will use the revised Figure files that have been submitted separately (these are clear and as per PLOS ONE guidelines)

********************************

**Reviewer #2:**

Comment

Point 1: In the result section, the first paragraph described the contents of table 2, not Table 1. Please check it carefully.

Response: Thanks for pointing our mistake. We have cross checked this and made corrections

Comment

Point 2: The contents of table 2 was more suitable described firstly in the Quantitative phase, the author should set table 2 to table 1.

Response: Thanks for suggestion. We discussed your comments and the authors decided to have the following order. To maintain the flow of the results section, we have first described the number (proportion) that were clinically assessed (each criteria separately). Then we mention the number (proportion) that undergo assessment for microbiological confirmation and how many TB patients (microbiologically confirmed and clinically confirmed) were eventually diagnosed. Then we talk about the treatment initiation and outcome. We hope this is fine. The tables and figures have been cited accordingly in the results narrative.

Comment

Point 3: In the Quantitative phase section, the description of tables and figures is illogical. The author should describe them as following figuere1, table2 and table1.

Response: Thanks for suggestion. We made the change. We have started with citing Figure 1. In line with our comments above, we have first described the table with baseline characteristics and clinical assessment. Then we have described the table depicting assessment of microbiological confirmation and TB diagnosis (microbiologically and clinically confirmed). (Line 247-257 in Revised Manuscript with tract changes)

Comment

Point 4: We still could not find “AFP” and “SSM” in the footnote of figure 1. Please check it carefully.
Response: Thanks for pointing our mistake. We removed “SSM” and corrected AFP to “AFB”. (Fig 1 footnotes)

Comment

Point 5: The image of figure1 and figure2 was blurry. Please use a higher definition image.

Response: Thanks for pointing out. The image files (Fig 1 and 2) are submitted separately at the time of submission. We have made sure that the image files are clear and meet PLOS ONE requirement. When these image files are merged into the pdf (while the software generates the submission pdf file), it is not uncommon for the figures to become blurred. However, as this comment has been made for the second time by the reviewer, we have remade the figures using MS powerpoint and submitted the more clear image files as figure files. (previously we used insert canvas option in MS word to make the figure files). We have taken this opportunity to make minor edits in Fig 1 and 2 so that they are consistent with the narrative.

Once accepted, the journal editorial office will use the revised Figure files that have been submitted separately (these are clear and as per PLOS ONE guidelines)
